# ASCT2 palmitoylation regulated by JNK1-ZDHHC14 axis orchestrates glutamine metabolism and NSCLC progression

**DOI:** 10.1038/s41421-026-00870-z

**Published:** 2026-02-24

**Authors:** Xingyu Chen, Zihao Ke, Shihui Wei, Jiajin Chen, Ke Zhu, Jiaqi Xu, Yun Zhao, Mengyuan Cen, Yan Jin, Zhilei Pan, Juan Xiong, Ying Chen, Chenfang Dong, Qianhua Cao, Chao Cao

**Affiliations:** 1 Key Laboratory of Respiratory Disease of Ningbo, Department of Respiratory and Critical Care Medicine, The First Affiliated Hospital of Ningbo University, Ningbo, Zhejiang China; 2 Institute of Engineering Medicine, The First Affiliated Hospital of Ningbo University, Ningbo, Zhejiang China; 3 Department of Pathology and Pathophysiology, and Department of Surgical Oncology (breast center), Key Laboratory of Cancer Prevention and Intervention, Ministry of Education, The Second Affiliated Hospital, Zhejiang University School of Medicine, Hangzhou, Zhejiang China

**Keywords:** Non-small-cell lung cancer, Post-translational modifications, Post-translational modifications

## Abstract

S-palmitoylation, a reversible post-translational modification regulates protein stability and cellular functions, yet its role in glutamine metabolism remains unclear. Here, we show that ZDHHC14 as the key palmitoyltransferase catalyzing ASCT2 palmitoylation at conserved Cys39 and Cys48 residues, promoting lysosomal degradation of this glutamine transporter, whereas ABHD17B functions as a depalmitoylase to stabilize ASCT2. Mechanistically, glutamine deprivation activates JNK1, which directly phosphorylates ZDHHC14 at Thr440 residue, triggering its degradation and thereby enhancing ASCT2 stability. Importantly, combination of JNK and ASCT2 inhibitors synergistically inhibits glutamine metabolism and tumor growth in vivo. These findings reveal a phosphorylation-palmitoylation axis linking JNK-mediated ASCT2 palmitoylation and glutamine metabolism, offering a potential therapeutic strategy for non-small cell lung cancer.

## Introduction

Non-small cell lung cancer (NSCLC) cells have a high dependency on glutamine to support cell mass accumulation and mitotic cell division by the KEAP1/NFE2L2 mutations, MYC amplification, or oncogenic KRAS^1,2^. Blocking glutamine metabolism has the potential to be a clinical strategy for the treatment of NSCLC. ASCT2, encoded by SLC1A5, is a sodium-coupled antiporter of neutral amino acids, mediates the exchange of amino acid substrates, and accounts for glutamine transport^3^. Aberrant ASCT2 expression has been implicated in highly proliferative cancer cells to fulfill enhanced glutamine demand and is associated with an aggressive phenotype and poor prognosis, addiction to ASCT2 in tumors has thus emerged as a significant target during the carcinogenesis process^4,5^.

S-palmitoylation (hereinafter referred to as palmitoylation) is a reversible post-translational modification that is catalyzed by the ZDHHC family of palmitoyltransferases and reversed by depalmitoylases^6,7^. Some studies have shown that palmitoylation is closely linked to the regulation of membrane localization, subcellular trafficking, and protein degradation in numerous cancers^8,9^. There is growing evidence for the involvement of palmitoylation in the regulation of multiple metabolic processes. For instance, ZDHHC9-mediated GLUT1 palmitoylation is critical for glucose supply during glioblastoma tumorigenesis^10^. ZDHHC20-dependent palmitoylation of FASN results in the inhibition of FASN ubiquitination to affect fatty acid metabolism in hepatocellular carcinoma^11^. However, the molecular mechanisms of palmitoylation regulating glutamine metabolism remain poorly understood.

The c-Jun N-terminal kinase (JNK), with its members JNK1, JNK2, and JNK3, as a subfamily of mitogen-activated protein kinase (MAPK), plays a critical role in cellular responses to multi-factorial stress^12^. A recent study identified that glutamine restriction induces ER stress to trigger an mTORC1-dependent increase in JNK activity^13^. Furthermore, JNK is frequently activated in NSCLC and directly phosphorylates proteins after activation, ultimately regulating processes such as apoptosis, cell proliferation, differentiation, and survival^14^. These observations underscore an essential role for JNK activation in NSCLC, and it has been validated as a potential therapeutic target for tumor progression^15^.

In the current study, we identify ASCT2 palmitoylation at conserved Cys39 and Cys48 residues, catalyzed by ZDHHC14 and reversed by ADHD17B, as a post-translational modification (PTM)-driven mechanism that regulates protein stability of ASCT2. Downregulation of ZDHHC14 expression in NSCLC promotes ASCT2 stabilization, leading to a high level of glutamine, thereby promoting NSCLC tumorigenesis. We further found that JNK1 phosphorylates ZDHHC14 at Thr440 and reduces ZDHHC14 stability to inhibit ASCT2 palmitoylation under glutamine deprivation. The inhibitor of JNK significantly synergizes with anti-ASCT2 therapy to retard tumor progression. By these efforts, we aim to bring additional insights on linking palmitoylation and glutamine metabolism that may provide new therapeutic strategies for targeting glutamine-dependent NSCLC.

## Results

### ZDHHC14 promotes ASCT2 palmitoylation and lysosome degradation

**Fig. 1:**
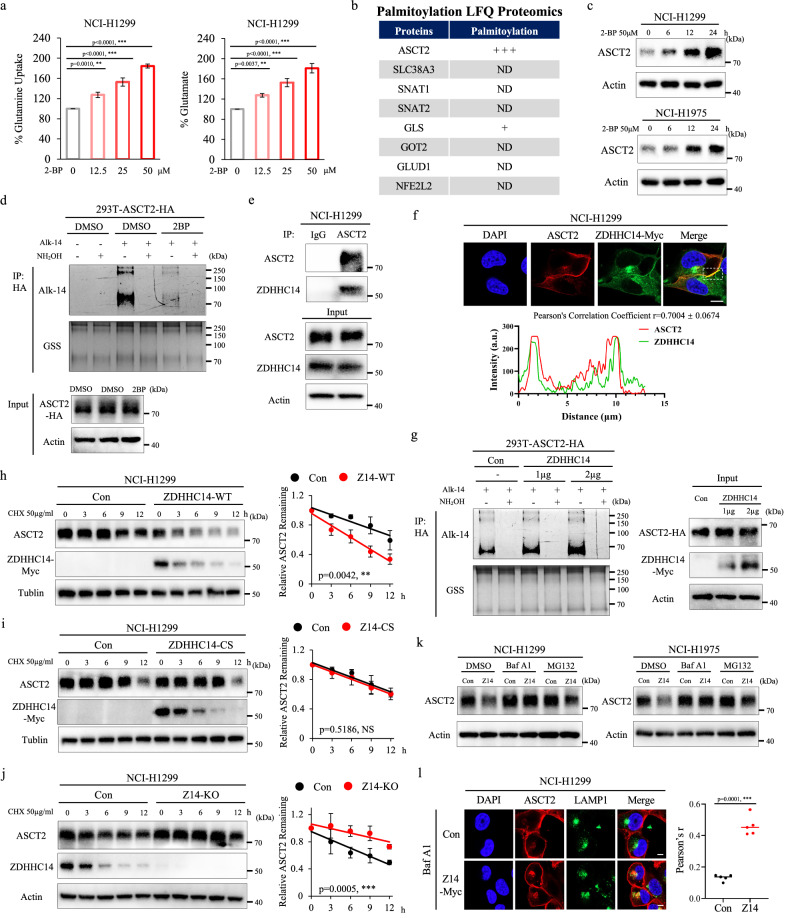
ZDHHC14 promotes ASCT2 palmitoylation and lysosome degradation. **a** NCI-H1299 cells were cultured with 2-BP at the indicated concentrations for 24 h. The media were collected for analysis of glutamine uptake (left), and the cells were collected for analysis of glutamate production(right). **b** Table showing the palmitoylations of glutamine metabolism regulators identified by Palmitoylation LFQ Proteomics. **c** NCI-H1299 (top) and NCI-H1975 (bottom) cells were treated with 50 μM 2-BP at the indicated time and subjected to western blot with ASCT2 and Actin antibodies. **d** ASCT2-HA expression HEK293T cells were cultured in medium containing 50 μM 2-BP and Alk14 for 8 h, and the cell lysates were prepared for immunoprecipitation and Alkyne-azide click chemistry experiments. Palmitoylation levels of ASCT2 with or without hydroxylamine (NH_2_OH) treatment were detected using Alk14 labeling by in-gel fluorescence detection, with silver staining the same gel to ensure consistent loading. **e** NCI-H1299 cells were harvested for co-IP assays using anti-IgG or anti-ASCT2 antibodies and then analyzed by western blot. **f** Exogenous ASCT2 and ZDHHC14 were stained by immunofluorescence assay in NCI-H1299 cells. DAPI, nucleus. Scale bar = 10 μm. Intensity traces (yellow lines) were plotted below. **g** HEK293T cells were co-transfected with ASCT2-HA and increasing amounts of ZDHHC14-Myc plasmid, immunoprecipitation and Alkyne-azide click chemistry experiments tested the cysteine palmitoyltransferase activity of ZDHHC14 on ASCT2. **h**, **i** NCI-H1299 cells with ZDHHC14-Myc (**h**) or catalytically inactive mutant ZDHHC14^C195S^-Myc (**i**) overexpression were incubated with 50 μg/mL cycloheximide (CHX) in a time-course frame, respectively. The collected cell lysates were subjected to immunoblotting detection with ASCT2, Myc, and Actin antibodies. The band density of ASCT2 was quantified and normalized to Actin (mean ± SD, *n* = 3). The *p* value was determined by two-way ANOVA. **j** NCI-H1299 ZDHHC14-knockout cells were treated with 50 μg/mL CHX for the indicated time. The collected cell lysates were subjected to western blot with ASCT2, Myc, and Actin antibodies. The band density of ASCT2 was quantified and normalized to Actin (mean ± SD, *n* = 3). The *p* value was determined by two-way ANOVA. **k** NCI-H1299 (left) and NCI-H1975 (right) cells with stable empty vector or ZDHHC14 expression were incubated with DMSO, 0.1 μM bafilomycin A1 (BafA1) or 10 μM MG132 for 6 h. The collected cell lysates were measured using ASCT2 and Actin antibodies. **l** Representative immunofluorescence pictures showing the co-localization of endogenous ASCT2 and lysosome-associated membrane protein-1 (LAMP1) in NCI-H1299 cells treated with BafA1, with statistical results shown on the right. Values are mean ± SD from five images (*n* = 5). The *p* value was determined by two-tailed Student’s *t*-test. DAPI, nucleus. Scale bar = 10 μm.

Protein palmitoylation is considered to have essential roles in various metabolic processes^6^. To investigate the multiple regulatory mechanisms of palmitoylation regulating glutamine metabolism, we treated cells with 2-bromopalmitate (2-BP), a general palmitoylation inhibitor, and performed glutamine uptake assays and glutamate production assays, showing that 2-BP significantly enhanced glutamine uptake and glutamate production in a dose-dependent manner (Fig. 1a). Following this lead, we performed palmitoylation proteomics analysis on NSCLC cell line to analyze the palmitoylation of glutamine metabolism regulators, showing that ASCT2 might be palmitoylated (Fig. 1b). To verify our hypothesis, we first tested ASCT2 protein level in NCI-H1299 and NCI-H1975 cells treated with palmitic acid sodium (palm), and found that ASCT2 protein level but not mRNA level was significantly decreased in a time-dependent manner (Supplementary Fig. S1a). Oppositely, inhibited palmitoylation by 2-BP increased the protein but not mRNA expression of ASCT2 in a time-dependent manner, suggesting that 2-BP regulates ASCT2 expression at the palmitoylation level (Fig. 1c; Supplementary Fig. S1b). Having established the effects of palm and 2-BP on ASCT2 protein levels, we next determined whether ASCT2 palmitoylation in HEK293T cells could be labeled by Alkyne-azide click chemistry experiments. Alkyne 14 (Alk14) as chemical reporter for palmitoylation, performed click chemistry to conjugate Alk14-labeled proteins with TAMRA azide, and determined the palmitoylation levels by fluorescence detection with silver stain the same gel to ensure consistent loading. Due to S-palmitoylation is sensitive to hydroxylamine, we found that the majority of the palmitoylation signal on ASCT2 was removed by treatment with hydroxylamine, suggesting ASCT2 is palmitoylated on cysteine, and 2-BP greatly reduced the level of palmitoylation (Fig. 1d). These findings suggested that ASCT2 palmitoylation destabilizes the ASCT2 protein in NSCLC.

Protein palmitoylation is catalyzed by the ZDHHC family of protein palmitoyltransferases. To screen the palmitoyltransferases responsible for ASCT2 palmitoylation, we transfected a series of plasmids encoding ZDHHC family members and assessed their interactions with ASCT2 by co-immunoprecipitation (Co-IP) assays. This analysis revealed that fourteen palmitoyltransferases (ZDHHC2, ZDHHC3, ZDHHC5, ZDHHC9, ZDHHC11, ZDHHC14, ZDHHC15, ZDHHC16, ZDHHC17, ZDHHC18, ZDHHC19, ZDHHC20, ZDHHC23 and ZDHHC24) specifically interacted with ASCT2 (Supplementary Fig. S1c). Next, we evaluated the effect of these palmitoyltransferases on ASCT2 palmitoylation by Alkyne-azide click chemistry experiments. As shown in Supplementary Fig. S1d, only ZDHHC14 but not the other thirteen candidates remarkably increased the palmitoylation signals. Subsequently, both exogenous and endogenous Co-IP assays results indicated that ZDHHC14 strongly interacted with ASCT2 (Fig. 1e; Supplementary Fig. S2a). Consistent with Co-IP assays, immunofluorescence analysis further confirmed a strong interaction between ASCT2 and ZDHHC14 at the plasma membrane of NCI-H1299 cells (Fig. 1f). Importantly, we detected that ZDHHC14 increased the palmitoylation signals in a dose-dependent manner (Fig. 1g). In contrast, ASCT2 palmitoylation was hardly detected in ZDHHC14-knockout cells (Supplementary Fig. S2b). Moreover, we found that 2-BP treatment inhibited ZDHHC14-mediated ASCT2 palmitoylation by Alkyne-azide click chemistry experiments (Supplementary Fig. S2c). Therefore, these data indicated that ZDHHC14 specifically regulates ASCT2 palmitoylation.

To further determine the effect of ZDHHC14-catalyzed palmitoylation on ASCT2, we examined ASCT2 protein stability in wild-type (WT) ZDHHC14, catalytically inactive ZDHHC14 mutant, or ZDHHC14-knockout cells. Following treating with protein synthesis inhibitor cycloheximide (CHX), the half-life of ASCT2 was markedly shortened in WT ZDHHC14-expressing cells, whereas catalytically inactive ZDHHC14 mutant (ZDHHC14^C195S^) only led to a slight change and ZDHHC14-knockout cells extended the half-life of ASCT2 (Fig. 1h–j; Supplementary Fig. S2d, e). Furthermore, immunoblot data showed that expression of WT ZDHHC14 but not the catalytically inactive ZDHHC14 mutant caused a remarkable decrease in ASCT2 protein level (Supplementary Fig. S2f). These data demonstrated that ASCT2 degradation is regulated by ZDHHC14, further reinforcing the enzyme–substrate relationship between ASCT2 and ZDHHC14 and the importance of palmitoylation modification.

The main mechanisms of protein degradation include the ubiquitin–proteasome pathway and lysosomal degradation pathway^16^. It has been reported that ZDHHC2 mediated the B-RAF and C-RAF palmitoylation to affect their autophagic degradation and stabilizing protein levels^17^. To further confirm the mechanisms of ZDHHC14-mediated ASCT2 degradation, we then treated the ZDHHC14 overexpression cells with lysosome inhibitor bafilomycin A1 (BafA1) and protease inhibitor MG132, and found that ZDHHC14-mediated ASCT2 degradation was blocked by BafA1 treatment, but not by MG132 treatment, suggesting that palmitoylation-mediated ASCT2 degradation is mainly regulated by lysosomal degradation pathway (Fig. 1k). Similar results were obtained in these cells by immunofluorescence analysis that ZDHHC14 overexpression promoted ASCT2 cytoplasmic localization and resulted in a change of ASCT2 lysosomal localization, showing a dramatic shift from diffuse to a focal perinuclear clustering in the lysosomal staining pattern and increased the co-localization of ASCT2 with lysosomal membrane protein LAMP1 (Fig. 1l; Supplementary Fig. S2g).

### ASCT2 is palmitoylated by ZDHHC14 at Cys39 and Cys48 sites

**Fig. 2:**
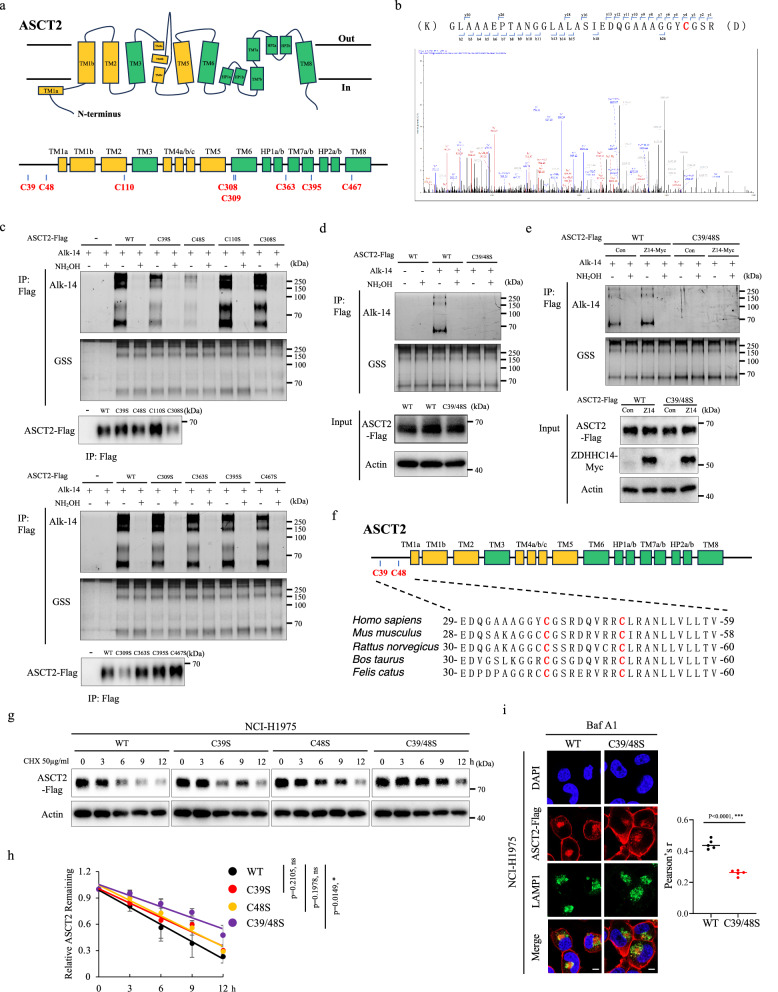
Cys39 and Cys48 sites of ASCT2 palmitoylation are mainly involved in lysosomal-mediated protein degradation. **a** Schematic diagram showing the ASCT2 structure with multiple transmembrane domains and eight possible palmitoylation sites. **b** LC-MS/MS spectra of representative ASCT2 peptides carrying palmitoylated Cys39 residue. **c** Expression of empty vector, WT ASCT2, or each cysteine residue mutation in HEK293T cells lysates was tested by immunoprecipitation, and Alkyne-azide click chemistry experiments. Palmitoylation levels of ASCT2 with or without hydroxylamine (NH_2_OH) treatment were detected by in-gel fluorescence with silver staining the same gel. **d** WT-ASCT2 or ASCT2^C39S/C48S^ mutants were expressed in HEK293T cells, and their palmitoylation levels were determined by Alkyne-azide click chemistry experiments. **e** ZDHHC14-mediated palmitoylation of WT or C39S/C48S mutant ASCT2 was assessed in HEK293T cells by immunoprecipitation and Alkyne-azide click chemistry. **f** Schematic diagram showing that palmitoylation sites of ASCT2 with conserved cysteine residues in *Homo sapiens*, *Mus musculus*, *Rattus norvegicus*, *Bos taurus*, and *Felis catus*. **g**, **h** NCI-H1975 cells stably expressing WT ASCT2 or various ASCT2 mutants were treated with 50 μg/mL CHX for the indicated time, and then the expression level of ASCT2 was analyzed by western blot (**g**). The band density of ASCT2 was quantified and normalized to Actin (mean ± SD, *n* = 3) (**h**). The *p* value was determined by two-way ANOVA. **i** Localization of ASCT2 and LAMP1 was measured by immunofluorescent staining in WT-ASCT2 or ASCT2^C39S/C48S^ mutant expression NCI-H1975 cells treated with BafA1, with statistical results shown on the right. Values are mean ± SD from five images (*n* = 5). The *p* value was determined by two-tailed Student’s *t*-test. Nuclei were visualized with DAPI (blue). Scale bar = 10 μm.

The protein structure of ASCT2 is characterized by a complex membrane-bound architecture with multiple transmembrane domains that facilitate its role as a Na^+^ dependent amino acid transporter^18^. ASCT2 protein consists of a polypeptide chain of 541 amino acids, and contains eight cysteine residues in total (Fig. 2a). The palmitoylation liquid chromatography-mass spectrometry (LC-MS/MS) analysis showed that Cys39 site of ASCT2 was palmitoylated (Fig. 2b; Supplementary Fig. S3a), whereas some peptides were too short for confident identification using standard LC-MS/MS because of the multiple tryptic cleavage sites (Arg/Lys) around the cysteine sites. To unambiguously identify the sites of ASCT2 palmitoylation, we systematically mutated each cysteine residue to serine in ASCT2 protein and tested their palmitoylation levels by Alkyne-azide click chemistry experiments, showing that ASCT2^C39S^ and ASCT2^C48S^ mutants both remarkably reduced ASCT2 palmitoylation, whereas mutating other sites singly (C110S, C308S, C309S, C363S, C395S, C467S) had a minor effect (Fig. 2c). Meanwhile, expression of ASCT2^C39S/C48S^ mutation completely eliminated the signals of ASCT2 palmitoylation, suggesting that Cys39 and Cys48 sites are essential for ASCT2 palmitoylation (Fig. 2d). Moreover, exogenous ZDHHC14 expression significantly promoted the level of palmitoylation in WT ASCT2 expression but not ASCT2^C39S/C48S^ mutant expression cells, whereas ZDHHC9, which is critical for glucose supply^10^, strongly interacting with ASCT2 in Supplementary Fig. 1c, did not affect the palmitoylation level, indicating that ZDHHC14 specifically mediates ASCT2 palmitoylation at Cys39 and Cys48 sites (Fig. 2e; Supplementary Fig. S3b).

Notably, the palmitoylation sites at Cys39 and Cys48 of ASCT2 are located in the N-terminal and highly evolutionarily conserved among different species (Fig. 2f). To analyze the effect of Cys39 and Cys48 sites on ASCT2 degradation, we treated WT ASCT2 or ASCT2^C39S^ mutant, ASCT2^C48S^ mutant and ASCT2^C39S/C48S^ mutant expression cells with protein synthesis inhibitor CHX, and found that the half-life of ASCT2 palmitoylation mutant cells was markedly extended compared with the WT ASCT2, resembling the effect of 2-BP treatment (Fig. 2g, h; Supplementary Fig. S3c). ASCT2 protein stability is known to be associated with its ubiquitination^19^, while overexpression of ASCT2 and palmitoylation mutants had a minor effect on ubiquitination levels with MG132 or BafA1 treatment (Supplementary Fig. S3d, e). These findings are consistent with a recent report showing that a neuron-specific protein, Ncdn, was localized to Rab5-positive early endosomes in a N-terminal-palmitoylated-dependent manner^20^. Following this lead, we examined the effect of palmitoylation on ASCT2 localization by immunofluorescence analysis, showing that ASCT2^C39S/C48S^ mutant significantly decreased the co-localization of LAMP1-positive lysosomal clusters in NCI-H1975 cells (Fig. 2i). As a result, Cys39 and Cys48 sites of ASCT2 palmitoylation are mainly involved in lysosomal-mediated protein degradation.

### ABHD17B depalmitoylates ASCT2 and promotes ASCT2 stabilization

**Fig. 3:**
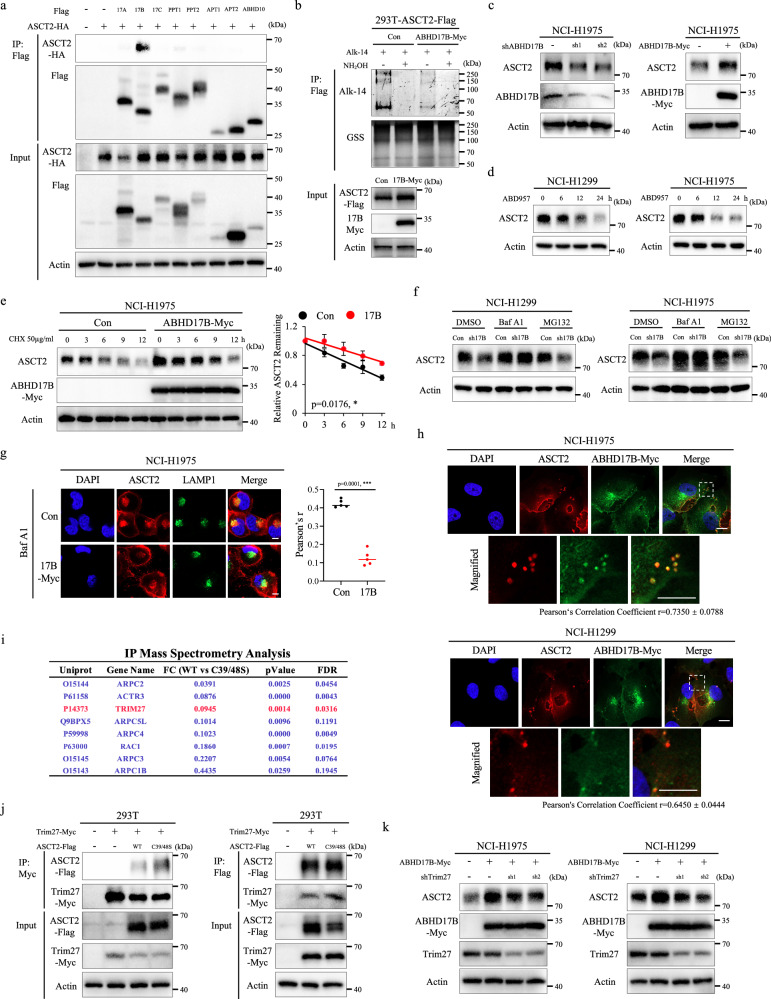
ABHD17B depalmitoylates ASCT2 and promotes ASCT2 stabilization. **a** ASCT2 was co-expressed with eight known depalmitoylases plasmid in HEK293T cells, and their interactions were detected by co-IP analyses. **b** ASCT2-Flag expression HEK293T cells were transfected with empty vector or ABHD17B-Myc plasmid, immunoprecipitation and Alkyne-azide click chemistry experiments tested the depalmitoylase activity of ABHD17B on ASCT2. **c** Expression of ASCT2 and ABHD17B was analyzed by western blot in NCI-H1975 cells with stable knockdown of ABHD17B expression (left) or ABHD17B overexpression (right). **d** NCI-H1299 (left) and NCI-H1975 (right) cells were treated with 1 μM ABD957 for the indicated time and subjected to western blot with ASCT2 and Actin antibodies. **e** NCI-H1975 cells with stable empty vector or ABHD17B expression were treated with 50 μg/mL CHX for the indicated time, and then the expression level of ASCT2 was analyzed by western blot. The band density of ASCT2 was quantified and normalized to Actin (mean ± SD, *n* = 3). The *p* value was determined by two-way ANOVA. **f** NCI-H1299 (left) and NCI-H1975 (right) cells with stable empty vector or knockdown of ABHD17B expression were incubated with DMSO, 0.1 μM BafA1, or 10 μM MG132 for 6 h. The collected cell lysates were measured using ASCT2 and Actin antibodies. **g** Localization of ASCT2 and LAMP1 was measured by immunofluorescent staining in empty vector or ABHD17B expression NCI-H1975 cells treated with BafA1, with statistical results shown on the right. Values are mean ± SD from five images (*n* = 5). The *p* value was determined by two-tailed Student’s *t*-test. Nuclei were visualized with DAPI (blue). Scale bar = 10 μm. **h** Representative immunofluorescence pictures showing the co-localization of ASCT2 and ABHD17B in NCI-H1975 (top) and NCI-H1299 (bottom) cells treated with BafA1. Pearson’s Correlation Coefficient was shown below. DAPI, nucleus. Scale bar = 10 μm. **i** IP-MS analysis showing candidates with increased binding to ASCT2^C39S/C48S^ compared to WT-ASCT2 in NCI-H1975 cells. Arp2/3 complex components are shown in blue. **j** The lysates of HEK293T cells with Trim27-Myc or/and ASCT2-Flag expression were subjected to immunoprecipitation with Myc beads (left) or Flag beads (right), and then their interactions were analyzed by western blot. **k** NCI-H1975 (left) and NCI-H1299 (right) cells were co-transfected as indicated, and the expression of ASCT2, ABHD17B-Myc, and Trim27 was measured by western blot.

To identify the potential depalmitoylase of ASCT2, a set of known depalmitoylases, such as ABHD17A, ABHD17B, ABHD17C, PPT1, PPT2, APT1, APT2 and ABHD10 were overexpressed and tested by Co-IP assays, showing that only ABHD17B strongly interacted with ASCT2 (Fig. 3a). In Alkyne-azide click chemistry experiments, exogenous ABHD17B expression robustly reduced the level of ASCT2 palmitoylation (Fig. 3b). To investigate whether ABHD17B regulates ASCT2 protein stability, we generated stable transfectants with knockdown of ABHD17B expression or ABHD17B overexpression in NCI-H1299 and NCI-H1975 cells. Immunoblot data showed that knockdown of *ABHD17B* expression exhibited a significant reduction in ASCT2 protein level, whereas exogenous ABHD17B expression resulted in a dramatic increase (Fig. 3c; Supplementary Fig. S4a). To further understand the effect of ABHD17B as a depalmitoylase on ASCT2, we examined ASCT2 accumulation by ABD957 treatment, a potent and selective covalent inhibitor of ABHD17A/B/C, showing that ABD957 treatment caused a time-dependent decrease in ASCT2 protein levels (Fig. 3d). Furthermore, CHX assay confirmed that exogenous ABHD17B expression repressed ASCT2 degradation, therefore sufficiently improved its protein accumulation (Fig. 3e; Supplementary Fig. S4b). In addition, lysosome inhibitor BafA1, rather than protease inhibitor MG132 treatment, blocked ASCT2 degradation in knockdown of *ABHD17B* expression cells (Fig. 3f). Further ubiquitination assay revealed that ABHD17B-mediated ASCT2 stability by ubiquitination-independent pathway (Supplementary Fig. S4c, d). Immunofluorescence analyses also showed that ABHD17B expression decreased the co-localization of ASCT2 with lysosomes (Fig. 3g). Notably, we surprisingly found that ABHD17B and ASCT2 were not co-localized at the plasma membrane (Fig. 3h).

To gain insight into the potential mechanisms underlying ASCT2-palmitoylated changes, we performed IP-MS Analysis of NCI-H1299 cells undergoing expression of WT ASCT2 or ASCT2^C39S/C48S^ mutant. It has been reported that the retrieval and recycling of ASCT2 to the cell surface relies on retromer, a master conductor of endosomal recycling^21^. We first compared the binding partners of ASCT2^C39S/C48S^ mutant with WT ASCT2 by IP-MS analysis and found that eight of the top candidates with increased binding to ASCT2^C39S/C48S^ mutant were retromer-dependent endosomal recycling components (Fig. 3i). TRIM27 as E3 RING ubiquitin ligase, localizes to endosomes through interactions with the retromer complex and is required for nucleation of endosomal F-actin by the WASH regulatory complex, a known regulator of retromer-mediated transport^22^. In addition, TRIM27 was significantly elevated in NSCLC and tightly associated with poor survival (Supplementary Fig. S4e, f). Given the tight association of TRIM27 with retromer-mediated transport and NSCLC, we chose it as an example to characterize the regulatory mechanism of ASCT2 palmitoylation. To understand the association of ASCT2 palmitoylation with TRIM27, we determined the effect of WT ASCT2 or ASCT2^C39S/C48S^ mutant on the interaction by co-immunoprecipitation assays, showing that the interaction between ASCT2 and TRIM27 was increased following expression of ASCT2^C39S/C48S^ mutant (Fig. 3j). To corroborate our findings, we examined ASCT2 protein level in ABHD17B and knockdown of TRIM27 co-expressing cells and found that knockdown of *TRIM27* expression dramatically decreased the level of ABHD17B-mediated ASCT2 accumulation but had only a minor effect on its ubiquitination levels (Fig. 3k; Supplementary Fig. S4g). Consistent with these results, ABHD17B-mediated ASCT2 depalmitoylation enhances ASCT2 retromer-dependent endosomal recycling.

### Glutamine deprivation inhibits ASCT2 palmitoylation by regulating ZDHHC14 stabilization

**Fig. 4:**
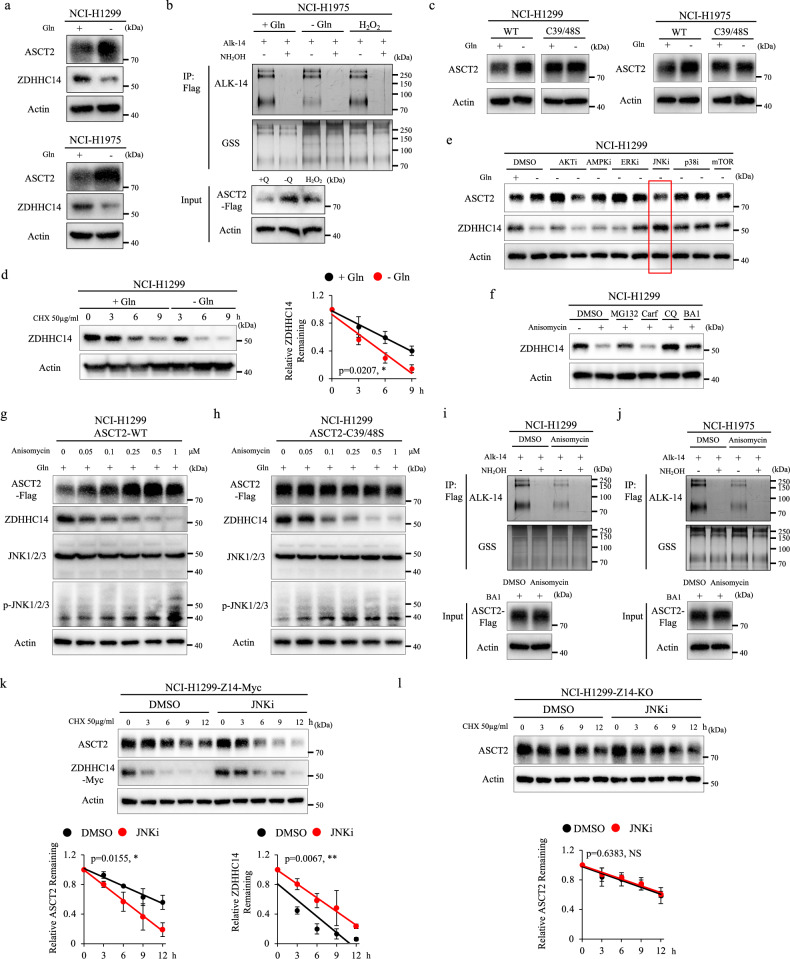
Glutamine deprivation inhibits ASCT2 palmitoylation through JNK pathway. **a** NCI-H1299 and NCI-H1975 cells were cultured in the medium with or without glutamine for 12 h, and then the expression levels of ASCT2 and ZDHHC14 were analyzed by western blot. **b** Palmitoylation effect of glutamine deprivation or H_2_O_2_ on ASCT2 in NCI-H1975 cells expressing ASCT2 was examined by immunoprecipitation (IP) and Alkyne-azide click chemistry experiments. **c** NCI-H1299 (left) and NCI-H1975 (right) cells expressing ASCT2-WT or ASCT2^C39S/C48S^ mutant were cultured in the medium with or without glutamine for 12 h, and the expression levels of ASCT2 were examined by western blot. **d** NCI-H1299 cells were cultured in the medium with or without glutamine and treated with addition of 50 μg/mL CHX, harvested at indicated time for western blot. The band density of ZDHHC14 was quantified and normalized to Actin (mean ± SD, *n* = 3). The *p* value was determined by two-way ANOVA. **e** Expression of ASCT2 and ZDHHC14 was analyzed by western blot in NCI-H1299 cells cultured in the medium with or without glutamine and treated with these indicated signaling pathway inhibitors (5 μM LY294002 and 5 μM AZD5363 for inhibiting AKT, 2 μM Dorsomorphin dihydrochloride for inhibiting AMPK, 5 μM Ravoxertinib and 5 μM SCH772984 for inhibiting ERK, 5 μM JNK-IN-8 for inhibiting JNK, 5 μM Adezmapimod and 5 μM SB-202190 for inhibiting p38, and 10 μM MHY1485 for activating mTOR) for 12 h. **f** NCI-H1299 cells were treated with DMSO, 10 μM MG132, 0.1 μM carf, 25 μM CQ or 0.1 μM BafA1 combined with 0.5 μM Anisomycin for 6 h. **g**–**i** ZDHHC14 expression was measured by western blot. NCI-H1299 cells expressing ASCT2-WT (**g**) or ASCT2^C39S/C48S^ mutant (**h**) were treated with 0.5 μM Anisomycin at the indicated concentrations for 12 h and subjected to western blot with ASCT2, ZDHHC14, JNK1/2/3, Phospho-JNK, and Actin antibodies. ASCT2-Flag expression NCI-H1299 (**i**) and NCI-H1975 cells (**j**) were treated with DMSO or Anisomycin, IP and Alkyne-azide click chemistry experiments examined the effect of Anisomycin on ASCT2 palmitoylation. **k** NCI-H1299 cells expressing ZDHHC14-Myc were treated with 50 μg/mL CHX and either DMSO or JNK inhibitor (JNK-IN-8), then harvested at indicated time for western blot. The band density of ASCT2 and ZDHHC14 was quantified and normalized to Actin (mean ± SD, *n* = 3). The *p* value was determined by two-way ANOVA. **l** ZDHHC14-knockout cells were co-treated with 50 μg/mL CHX and either DMSO or JNK inhibitor (JNK-IN-8), then harvested at indicated time for western blot. The band density of ASCT2 was quantified and normalized to Actin (mean ± SD, *n* = 3). The *p* value was determined by two-way ANOVA.

A previous study showed that ASCT2 is subjected to upregulation under glutamine deprivation^19^; however, the effect of palmitoylation on ASCT2 stability under this stressed condition has not been elucidated. To investigate the possible stressed or physiological conditions that would modulate the ASCT2 palmitoylation, we applied immunoblot analysis and found that the level of ASCT2 was dramatically increased under glutamine deprivation in NCI-H1299 and NCI-H1975 cells (Fig. 4a). Following this lead, we tested the signals of ASCT2 palmitoylation under glutamine deprivation by Alkyne-azide click chemistry experiments. As anticipated, glutamine deprivation conditions greatly decreased the signals of ASCT2 palmitoylation (Fig. 4b; Supplementary Fig. S5a). We further observed no significant difference for ASCT2 in ASCT2^C39S/C48S^ mutant expression cells (Fig. 4c). To explicitly demonstrate the role and mechanism of glutamine deprivation in ASCT2 palmitoylation, we then evaluated the effect on palmitoyltransferase-ZDHHC14 or depalmitoylase-ABHD17B. Interestingly, the protein level of ZDHHC14 was minimized under glutamine deprivation, while the interaction between ASCT2 and ABHD17B was decreased, further showing that only palmitoylated ASCT2 could bind to ABHD17B (Fig. 4a; Supplementary Fig. S5b). Consistently, cultured cells with CHX treatment further validated that glutamine deprivation promoted ZDHHC14 degradation in time-dependent manners in NCI-H1299 and NCI-H1975 cells (Fig. 4d; Supplementary Fig. S5c). These findings suggested that glutamine deprivation inhibits ASCT2 palmitoylation by reducing ZDHHC14 stabilization.

Previous studies have illustrated that nutrient stress caused by glutamine deprivation leads to the activation of p38-MAPK, Akt-mTOR, ERK, and JNK pathways^23–26^. To understand the molecular mechanisms underlying glutamine deprivation-mediated ZDHHC14 stability, we tested ZDHHC14 and ASCT2 protein levels combined with these signaling pathway inhibitors treatment under glutamine deprivation, showing that only JNK inhibitor treatment not only elevated the level of ZDHHC14 but also reduced the level of ASCT2 in NCI-H1299 and NCI-H1975 cells (Fig. 4e; Supplementary Fig. S5d). We noticed that p38 inhibitors also caused an accumulation of ZDHHC14 because p38 inhibitors treatment repressed lysosomal function as previously reported^27^. Furthermore, JNK agonist, Anisomycin, combined with autophagy inhibitors BafA1 and chloroquine (CQ) rather than proteasome inhibitor MG132 and Carfilzomib (Carf) restored the ZDHHC14 levels, suggesting that JNK-mediated ZDHHC14 degradation is mainly regulated by lysosomal degradation pathway (Fig. 4f; Supplementary Fig. S5e). Moreover, Anisomycin treatment remarkably up-regulated the level of ASCT2 with a decrease of ZDHHC14 in a dose-dependent manner (Fig. 4g; Supplementary Fig. S5f), whereas the level of ASCT2 in ASCT2^C39S/C48S^ mutant expression cells was not affected, indicating that the effects of JNK pathway on ZDHHC14-mediated ASCT2 stability were indeed dependent on palmitoylation-driven mechanism (Fig. 4h; Supplementary Fig. S5g). In line with the observations by immunoblot analysis, Alkyne-azide click chemistry experiments results indicated that Anisomycin markedly inhibited ASCT2 palmitoylation (Fig. 4i, j), whereas JNK inhibitor dramatically enhanced ASCT2 palmitoylation under glutamine deprivation (Supplementary Fig. S5h). Subsequently, we performed CHX chase assays and validated that JNK inhibitor extended ZDHHC14 half-life and promoted ASCT2 degradation in time-dependent manners in ZDHHC14-expressing cells, whereas JNK inhibitor only led to a slight change in ZDHHC14-knockout cells (Fig. 4k, l). Hence, these data underscored that JNK pathway as the major signaling pathway regulates ZDHHC14-mediated ASCT2 palmitoylation under glutamine deprivation.

### ZDHHC14 is phosphorylated by JNK1 at Thr440 in response to glutamine deprivation stimuli

**Fig. 5:**
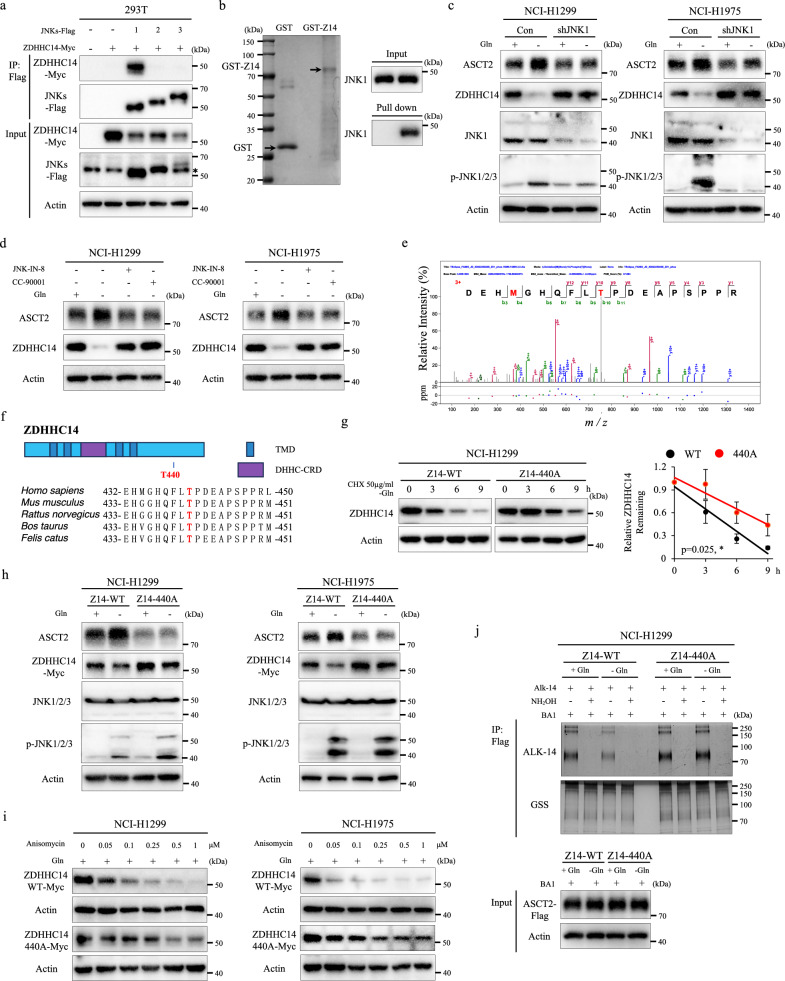
JNK1-mediated phosphorylation stabilizes ASCT2 by triggering ZDHHC14 degradation. **a** The lysates of HEK293T cells with ZDHHC14 and indicated JNK members expression were subjected to IP with Flag magnetic beads, and then their interactions were analyzed by western blot. **b** Representative immunoblotting bands for GST precipitation showing ZDHHC14-JNK1 binding by treating purified GST or GST-ZDHHC14 protein with purified 6His-JNK1 protein in vitro. **c** NCI-H1299 (left) and NCI-H1975 (right) cells were transfected with stable empty vector or knockdown of JNK1 expression, then cultured in the medium with or without glutamine for 12 h, the expression of ASCT2, ZDHHC14, JNK1, and p-JNK was measured by western blot. **d** NCI-H1299 (left) and NCI-H1975 (right) cells were treated with DMSO, 10 μM JNK-IN-8, or 10 μM CC-90001 with culturing in the medium with or without glutamine for 12 h, expression of ASCT2 and ZDHHC14 was measured by western blot. **e** MS analysis of representative ZDHHC14 peptides carrying Thr440 phosphorylation. **f** Schematic diagram showing that phosphorylation site of ZDHHC14 with conserved residue in *Homo sapiens*, *Mus musculus*, *Rattus norvegicus*, *Bos taurus*, and *Felis catus*. **g** For CHX chase assay, ZDHHC14-WT or phosphorylation mutant ZDHHC14^T440A^ was expressed in NCI-H1299 cells, and then cells were treated with 50 μg/mL CHX for the indicated time, the expression level of ZDHHC14 was analyzed by western blot. The band density of ZDHHC14 was quantified and normalized to Actin (mean ± SD, *n* = 3). The *p* value was determined by two-way ANOVA. **h** NCI-H1299 (left) and NCI-H1975 (right) cells were transfected with ZDHHC14-WT or ZDHHC14 phosphorylation mutant ZDHHC14^T440A^, then cultured in the medium with or without glutamine for 12 h, expression of ASCT2, ZDHHC14, JNK1/2/3, Phospho-JNK1/2/3 was measured by western blot. **i** NCI-H1299 (left) and NCI-H1975 (right) cells expressing ZDHHC14-WT or ZDHHC14 phosphorylation mutant ZDHHC14^T440A^ were treated with Anisomycin at the indicated concentrations for 12 h, and the expression of ZDHHC14 was analyzed by western blot. **j** NCI-H1299 cells were co-expressed with ASCT2-Flag and ZDHHC14-WT/ZDHHC14^T440A^ mutant, then cultured in the medium with or without glutamine for 12 h, immunoprecipitation and Alkyne-azide click chemistry experiments examined the palmitoylation level of ASCT2.

We next sought to determine the detailed mechanisms of JNK regulates ZDHHC14-mediated ASCT2 palmitoylation. JNK pathway, including three isoforms (JNK1, JNK2, and JNK3) with slicing variant, plays a critical role in cellular responses to a variety of stress signals^12^. We determined the interactions between ZDHHC14 and JNK isoforms by co-IP analyses, showing that only JNK1 and ZDHHC14 form a complex (Fig. 5a). Protein-protein docking analysis also revealed a protein interaction between ZDHHC14 and JNK1 (Supplementary Fig. S6a). Then, we overexpressed ZDHHC14 in *ZDHHC14*-knockout cells to mimic the WT cell line for Co-IP analyses, the results showed that ZDHHC14 strongly interacted with ASCT2 and JNK1 (Supplementary Fig. S6b). Furthermore, JNK1 could directly bind to GST-ZDHHC14 rather than to GST alone, which indicated the direct interaction between JNK1 and ZDHHC14 (Fig. 5b). To further define the potential effect of JNK1 on ZDHHC14, knockdown of JNK1 expression NCI-H1299 and NCI-H1975 cells were cultured in the medium with or without glutamine, showing that knockdown of JNK1 significantly elevated ZDHHC14 expression and inhibited ASCT2 expression under glutamine deprivation (Fig. 5c). Consistently, JNK-IN-8 and CC-90001 treatment, both inhibiting JNK1 phosphorylation and activation, caused ZDHHC14 accumulation and reduced ASCT2 level under glutamine deprivation (Fig. 5d).

To identify the amino acid residue(s) of ZDHHC14 that is phosphorylated in response to glutamine deprivation stimuli, we established an in vitro kinase assay combined with LC-MS/MS (Supplementary Fig. S6c). Interestingly, three phosphorylation sites of ZDHHC14 at Thr124, Thr440, and Ser455 were identified under stimulating with ATP (Fig. 5e, f; Supplementary Fig. S6c, d). To assess the phosphorylation site that influences ZDHHC14 protein stability, we examined the effect of glutamine deprivation conditions on ZDHHC14 protein levels in WT ZDHHC14 or non-phosphorylation mutant expression cells. Intriguingly, in contrast to WT ZDHHC14, only ZDHHC14^T440A^ mutant dramatically rescued the reduction of ZDHHC14 protein levels under glutamine deprivation, suggesting that Thr440 site is essential for ZDHHC14 protein stability (Supplementary Fig. S6e).

Moreover, WT ZDHHC14 or non-phosphorylation-mimetic ZDHHC14 mutant expression cells with CHX treatment revealed that ZDHHC14^T440A^ extended ZDHHC14 protein half-life in a time-dependent manner under glutamine deprivation (Fig. 5g; Supplementary Fig. S6f). Consistently, WT ZDHHC14 but not ZDHHC14^T440A^ mutant remarkably regulated both ZDHHC14 and ASCT2 stabilization, as well as ZDHHC14 phosphorylation in response to glutamine deprivation, while this regulatory effect was strongly inhibited by JNK inhibitor (Fig. 5h; Supplementary Fig. S7a, b). As can be seen in Fig. 5i, ZDHHC14^T440A^ also repressed ZDHHC14 degradation by Anisomycin treatment in a dose-dependent manner. Finally, we performed Alkyne-azide click chemistry experiments, showing that ZDHHC14^T440A^ mutant expression robustly restored the level of ASCT2 palmitoylation following glutamine deprivation or Anisomycin treatment (Fig. 5j; Supplementary Fig. S7c, d). Collectively, these data demonstrated that in response to glutamine deprivation, JNK1 is activated to phosphorylate ZDHHC14 at Thr440 residue that triggers ZDHHC14 degradation, leading to inhibit ASCT2 palmitoylation modification and subsequently raise ASCT2 accumulation.

### ZDHHC14-mediated ASCT2 palmitoylation regulates glutamine metabolism and tumorigenesis

**Fig. 6:**
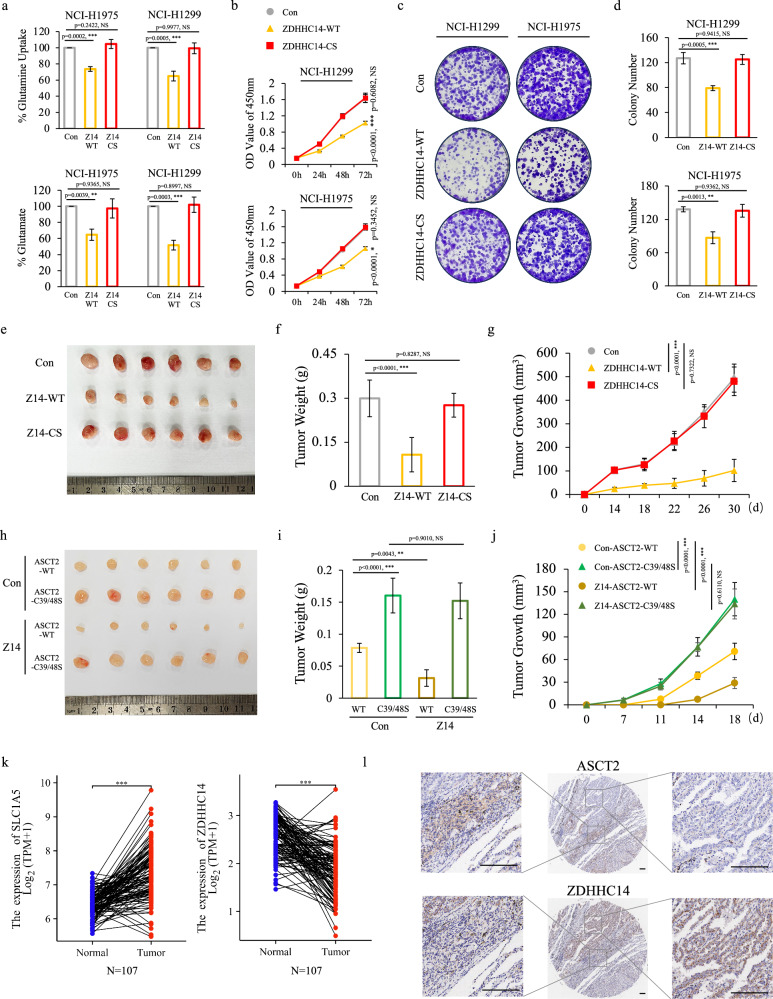
ZDHHC14-ASCT2 axis regulates glutamine metabolism and tumorigenesis in NSCLC. **a** Glutamine uptake and glutamate production in NCI-H1975 (left) and NCI-H1299 (right) cells with stable empty vector, ZDHHC14-WT or catalytically inactive mutant ZDHHC14^C195S^ expression were measured by assays for glutamine uptake (up) and glutamate production (bottom) detection (mean ± SD in three separate experiments). **b** Cell viability of the ZDHHC14-WT or catalytically inactive mutant ZDHHC14^C195S^ expressing cells was analyzed for the indicated times. Data were presented as a percentage of control cell lines (mean ± SD in three separate experiments). The *p* value was determined by two-way ANOVA. **c**, **d** Representative pictures showed colony formation of NCI-H1299 (left) and NCI-H1975 (right) cells expressing stable empty vector, ZDHHC14-WT, or catalytically inactive mutant ZDHHC14^C195S^ (**c**). Data were presented as a percentage of empty vector cell lines (mean ± SD in three separate experiments) (**d**). The *p* value was determined by one-way ANOVA. **e**–**g** NCI-H1299 cells expressing stable empty vector, ZDHHC14-WT or catalytically inactive mutant ZDHHC14^C195S^ were injected subcutaneously into the flanks of Balb/c nude mice. The growth of tumors was examined every 4 days. Images of subcutaneous xenografts from the indicated NCI-H1299 cells; *n* = 6 mice per group (**e**). Tumor size and weight were recorded (**f**, **g**). Data were shown as mean ± SEM from six mice. The *p* value of tumor size was determined by two-way ANOVA, the *p* value of tumor weight was determined by one-way ANOVA. **h**–**j** Images (**h**), weight (**i**), and tumor size (**j**) of subcutaneous xenografts from the indicated NCI-H1299 cells; *n* = 6 mice per group. Data were shown as mean ± SEM from six mice. The *p* value of tumor size was determined by two-way ANOVA, the *p* value of tumor weight was determined by one-way ANOVA. **k** Analysis of the TCGA dataset for the expression of ASCT2 (left) and ZDHHC14 (right). The *p* value was determined by paired *t*-test. **l** Representative images of ASCT2 and ZDHHC14 IHC staining in consecutive NSCLC tissues. Scale bar = 100 μm.

ASCT2, as a glutamine transporter, controls cellular uptake of glutamine and participates in the progression of tumors and metabolic diseases^28^. Therefore, we performed glutamine uptake assays and glutamate production assays to further determine the metabolic changes of ASCT2 palmitoylation. As expected, ZDHHC14 significantly suppressed glutamine uptake and glutamate production, whereas catalytically inactive ZDHHC14 mutant (ZDHHC14^C195S^) only caused a slight change in NCI-H1299 and NCI-H1975 cells (Fig. 6a). These results indicated that ZDHHC14, the palmitoyltransferase of ASCT2 palmitoylation, functions as an important factor of glutamine metabolism.

Having identified the critical association of ZDHHC14 with metabolic alteration in NSCLC, we sought to evaluate the functional role of ZDHHC14 in vitro and in vivo. We first examined the in vitro proliferation using growth and colony formation analyses, showing that WT ZDHHC14 significantly inhibited cell growth and colony formation in NCI-H1299 and NCI-H1975 cells, whereas catalytically inactive ZDHHC14 mutant (ZDHHC14^C195S^) only caused a minor effect (Fig. 6b–d). Similar results were obtained in vivo by tumor xenograft experiments in which male BALB/c nude mice were injected with stable empty vector, WT ZDHHC14 expression, or catalytically inactive ZDHHC14 mutant expression NCI-H1299 cells. As shown in Fig. 6e–g, injection of NCI-H1299 cells with stable WT ZDHHC14 expression but not catalytically inactive ZDHHC14 mutant expression resulted in significant inhibition of tumor growth in vivo. To further evaluate the effect of ZDHHC14-mediated ASCT2 Cys39 and Cys48 palmitoylation on tumorigenesis, NCI-H1299 cells co-expressing ZDHHC14 and WT ASCT2 or ASCT2^C39S/C48S^ mutant were injected subcutaneously into nude mice. As expected, cells expressing ASCT2^C39S/C48S^ mutant exhibited significantly increased tumor growth compared with cells expressing WT ASCT2. Notably, ZDHHC14 expression only inhibited tumor growth in WT ASCT2 expression group but not ASCT2^C39S/C48S^ mutant expression group (Fig. 6h–j).

Given the critical function of ZDHHC14-mediated palmitoylation in NSCLC, we hypothesized ZDHHC14 and ASCT2 expression was correlated with patient survival in clinical datasets. In support of this hypothesis, clinical data analysis showed that the mRNA levels of ZDHHC14 were significantly decreased in NSCLC tissues compared to those in normal tissues (Fig. 6k; Supplementary Fig. S8a). Moreover, NSCLC patients with lower ZDHHC14 expression levels had shorter survival, as illustrated by the Kaplan-Meier plot database (Supplementary Fig. S8b). In contrast, high expression of ASCT2 was closely correlated with NSCLC and linked to a dismal overall survival (Fig. 6k; Supplementary Fig. S8a–c). Consistent with bio-informatics data analysis, immunohistochemistry (IHC) staining analysis further confirmed that there was an inverse correlation between ASCT2 and ZDHHC14 (Fig. 6l; Supplementary Fig. S8d). Taken together, these analyses revealed that a high level ASCT2 expression negatively correlates with ZDHHC14 expression and is essential for clinical progression of NSCLC.

### Targeting JNK pathway promotes the anti-cancer effect of ASCT2 inhibitor V9302

**Fig. 7:**
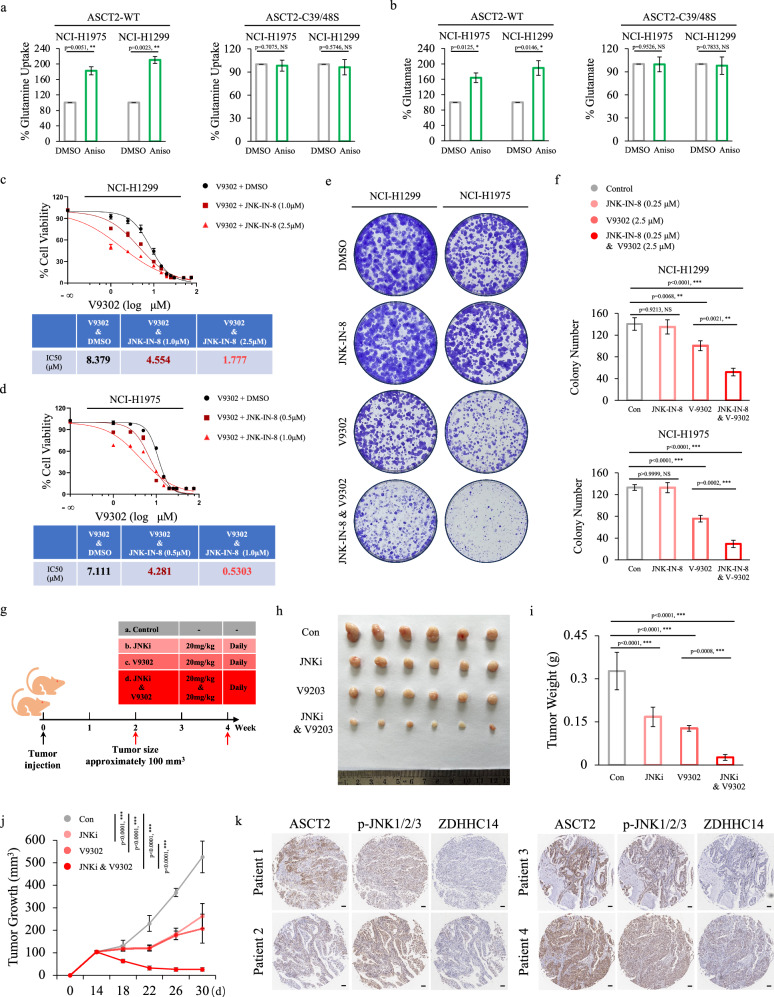
Synergistic anti-cancer activity of the combination of V9302 and JNKi in NSCLC. **a**, **b** NCI-H1975 and NCI-H1299 cells expressing WT-ASCT2 (left) or ASCT2^C39S-C48S^ mutant (right) were cultured with or without 1 μM Anisomycin treatment for 12 h. The media were collected for analysis of glutamine uptake (**a**) and the cells were collected for analysis of glutamate production (**b**) (mean ± SD in three separate experiments). The *p* value was determined by two-tailed Student’s *t*-test. **c**, **d** Cell viability of NCI-H1299 (top) and NCI-H1975 (bottom) cells was analyzed following treatment with indicated concentration of V9302 alone or with indicated concentration of JNK-IN-8 for 72 h, and the IC50 was calculated. **e**, **f** Colony formation assay was performed using NCI-H1299 (left) and NCI-H1975 (right) cells treating with DMSO, 0.25 μM JNK-IN-8, 2.5 μM V9302, alone or in combination. Representative colony formation images were shown (**e**). Data were presented as a percentage of cell lines with DMSO treatment (mean ± SD in three separate experiments) (**f**). The *p* value was determined by one-way ANOVA. **g** Timeline of the mouse model to evaluate the anti-cancer efficiency of JNK-IN-8, V9302, alone or in combination in vivo. **h**–**j** The tumor growth in vivo of NCI-H1299 xenograft tumors was examined with the treatment of JNK-IN-8 (20 mg/kg/day; i.p.), V9302 (20 mg/kg/day i.p.), alone or in combination for 16 days. The growth of tumors was examined every 4 days (**j**). Tumor size and weight were recorded (**i**, **j**). Data were shown as mean ± SEM from six mice. The *p* value of tumor size was determined by two-way ANOVA, the *p* value of tumor weight was determined by one-way ANOVA. **k** Representative images of ASCT2, Phospho-JNK, and ZDHHC14 IHC staining in consecutive NSCLC tissues. Scale bar = 100 μm.

Recent studies revealed that pharmacological blockade of ASCT2 with V9302 resulted in attenuating cancer cell growth and proliferation^29^. Given our observation that JNK1 phosphorylates ZDHHC14 to promote ZDHHC14 degradation, thereby enhancing palmitoylation-mediated ASCT2 stability, we hypothesized that targeting JNK pathway might synergize with anti-ASCT2 therapy to elicit an enhanced therapeutic effect. We first determined whether JNK activation resulted in a change of glutamine metabolism. As expected, JNK agonist, Anisomycin, dramatically promoted the glutamine metabolism in endogenously WT ASCT2-expressing cells but not ASCT2^C39S/C48S^ mutant-expressing cells (Fig. 7a, b), further supporting that JNK1 regulates glutamine metabolism by mediating ASCT2 palmitoylation. Meanwhile, based on the regulatory relationship between ABHD17B and ASCT2 palmitoylation, we also treated cells with ABD957, an inhibitor of ABHD17, and performed glutamine uptake assays and glutamate production assays, showing that ABD957 significantly suppressed the glutamine metabolism in NCI-H1299 and NCI-H1975 cells (Supplementary Fig. S9a, b). Subsequently, combination with JNK and ASCT2 inhibitor synergistically suppressed glutamine uptake and glutamate production (Supplementary Fig. S9c, d).

To further determine the functional consequences of JNK-mediated ASCT2 palmitoylation, we measured the IC50 values of JNK inhibitor JNK-IN-8 in NCI-H1299 and NCI-H1975 cells, and used the low-toxic IC10 values to determine V9302 sensitization (Supplementary Fig. S9e, f). As shown in Fig. 7c, d, JNK-IN-8 increased the sensitivity of NCI-H1299 and NCI-H1975 cells to V9302 in the tested concentrations. Moreover, the colony formation analyses showed that a combination of JNK inhibitor JNK-IN-8 and ASCT2 inhibitor V9302 caused greater inhibition of colony formation than either of compound used alone (Fig. 7e, f).

To evaluate the anti-cancer efficiency of these inhibitors in vivo, we injected NCI-H1299 cells in Balb/c nude mice and subsequently assessed tumor response to JNK-IN-8 treatment alone, V9302 treatment alone, or double pharmacological combination treatment. Two weeks after NCI-H1299 cells inoculation and when tumor volume reached approximately 100 mm^3^, mice were randomly allocated to receive: (1) vehicle, (2) JNK-IN-8 (20 mg/kg/day; i.p.), (3) V9302 (20 mg/kg/day i.p.), (4) JNK-IN-8 (20 mg/kg/day; i.p.) and V9302 (20 mg/kg/day i.p.) for 16 days (Fig. 7g). In accordance with our previous results in colony formation analyses, the anti-tumor effect in the group of combination with JNK and ASCT2 inhibitor was better than mono-therapy (Fig. 7h–j; Supplementary Fig. S9g). IHC staining analysis further confirmed a strong interaction between ASCT2, Phospho-JNK, and ZDHHC14 (Fig. 7k; Supplementary Fig. S8d). Collectively, these analyses revealed that the double pharmacological treatment targeting JNK and ASCT2 was synergistic in inhibiting tumorigenicity in vitro and in vivo, defining a rational drug combination for NSCLC clinical therapy.

## Discussion

Our study establishes that ASCT2 undergoes dynamic palmitoylation at evolutionarily conserved Cys39 and Cys48 residues, a modification catalyzed by the palmitoyltransferase-ZDHHC14. This lipid modification serves as a critical regulatory switch controlling ASCT2’s subcellular localization, directing ASCT2 to lysosomal degradation pathways, whereas depalmitoylation by ABHD17B stabilizes ASCT2 and promotes its plasma membrane localization for glutamine uptake. The balance between these opposing enzymatic activities creates a finely tuned system for regulating cellular glutamine transport capacity.

Another key regulatory mechanism we demonstrate that JNK1 phosphorylates ZDHHC14 at Thr440 residue under glutamine deprivation conditions, marking it for lysosomal degradation, and thereby reducing ASCT2 palmitoylation and increasing its stability. This phosphorylation-dependent regulation of ZDHHC14 stability creates a direct link between metabolic stress and ASCT2 palmitoylation status, representing a crucial adaptive mechanism that allows cancer cells to maintain glutamine uptake despite extracellular nutrient scarcity. This JNK1-ZDHHC14-ASCT2 axis effectively couples cellular metabolic state to glutamine transport capacity through dynamic PTMs, providing cancer cells with a growth advantage.

Cancer cells highly depend on glutamine metabolism to fuel tumor growth and proliferation^30^. This metabolic vulnerability induces microenvironmental glutamine scarcity through accelerated consumption, consequently activating JNK pathway as an adaptive stress response mechanism. Notably, KEAP1 or NFE2L2 mutations are frequently observed in NSCLC and drive tumor progression, conferring heightened glutamine dependence to NSCLC cells for survival and proliferation^1^. Our study reveals a promising therapeutic strategy for targeting glutamine-dependent cancers. The synergistic anti-tumor effects observed with combined JNK and ASCT2 inhibition suggest that simultaneously targeting both the upstream regulator (JNK) and downstream effector (ASCT2) of this pathway may be particularly effective against glutamine-dependent tumors. Clinically, the inverse correlation between ZDHHC14 and ASCT2 expression could serve as a biomarker for patient stratification, indicating low ZDHHC14/high ASCT2 expression in tumors may represent a subset particularly vulnerable to therapies that disrupt this regulatory axis. Additionally, our findings open new avenues for developing small-molecule modulators of ZDHHC14 or ABHD17B activity as potential anti-cancer agents, and suggest that manipulating protein palmitoylation may represent a novel strategy to disrupt cancer metabolism more broadly.

In summary, our study reveals a novel phosphorylation-palmitoylation cascade linking nutrient sensing to glutamine transport in NSCLC. We demonstrate how JNK1-mediated ZDHHC14 phosphorylation controls ASCT2 palmitoylation and stability, uncovering a metabolic adaptation mechanism. These findings establish palmitoylation as a key regulatory node in cancer metabolism and highlight the JNK1-ZDHHC14-ASCT2 axis as a potential therapeutic target.

## Materials and methods

### Chemicals

JNK-IN-8 (#HY-13319), CC-90001 (#HY-138304), MG132 (#HY-13259), ABD957 (#HY-142161), 2-Bromohexadecanoic acid (2-BP) (#HY-111770), Bafilomycin A1 (BafA1) (#HY-100558), Chloroquine (CQ) (#HY-17589A), Carfilzomib (Carf) (#HY-10455), V9302 (#HY-112683 & #HY-112683A), LY294002 (#HY-10108), AZD5363 (#HY-15431), Ravoxertinib (#HY-15947), SCH772984 (#HY-50846), Adezmapimod (#HY-10256), SB-202190 (#HY-10295), Dorsomorphin dihydrochloride (#HY-13418) and MHY1485 (#HY-B0795) were purchased from MedChemExpress. Palmitic acid sodium (#E2900) was purchased from Selleck. Palmitic Acid Alkyne (#13266) was purchased from Cayman Chemical. Hydroxylamine solution (#438227), Tris ((1-benzyl-1H-1,2,3-triazol-4-yl) methyl) amine (TBTA, #678937), and tris (2-carboxyethyl) phosphine HCl (TCEP, #C4706) was purchased from Sigma-Aldrich. TAMRA azide, 5-isomer (#LMP-C7130) was purchased from Lumiprobe. Puromycin (#ST551) and G418 (#ST081) were purchased from Beyotime.

### Cell lines

HEK293T cells and human LUAD cells (NCI-H1299, NCI-H1975) were obtained from the Procell Life Science & Technology Co., Ltd. HEK293T cells were cultured in Dulbecco’s modified Eagle’s Medium (DMEM) supplemented with 10% FBS. NCI-H1299 and NCI-H1975 were cultured in RPMI-1640 medium supplemented with 10% FBS. All the cells were cultured and stored according to the instruction from the ATCC. For establishing stable transfectants with overexpression of WT or mutated ASCT2, overexpression of WT or mutated ZDHHC14, overexpression or knockdown expression of ABHD17B, stable clones were selected using 200 ng/mL puromycin for 3–4 weeks.

### Stable cell line generation

To harvest lentivirus containing shRNA against ABHD17B, JNK1, and Trim27, HEK293T cells were transfected with shRNA-expressing plasmids (pLKO-1, Sigma-Aldrich), together with psPAX2 and pMD2G. The sequences of shRNAs are as follows: shABHD17B: 5′-TGATCCAACTTACACACTGAT-3′, 5′-CTTGGTCAAATGAGCAGCTTT -3′; shJNK1: 5′-CGGGCTGTTCTCCTGCATCAT-3′; shTrim27: 5′-GCCCTACTTCAGTCTGAGTTA-3′, 5′-GAGAAGATTGTTTGGGAGTTT-3′. ZDHHC14-knockout NCI-H1299 cells were generated using CRISPR/Cas9, sgRNA (5′-ATCGGCGGCTTCATCAGGCGTGG-3′) targeting ZDHHC14 was cloned into LentiCRISPRv2, and lentivirus was produced in HEK293T cells. After infection and puromycin selection of NCI-H1299, single-cell clones were isolated by limiting dilution. Knockout was confirmed by sequencing and western blot. To harvest lentivirus expressing WT ZDHHC14, mutant ZDHHC14, WT ASCT2, and mutant ASCT2, HEK293T cells were transfected with constructs containing cDNA in pLVX-puro or pLVX-Neo-IRES (Takara), together with psPAX2 and pMD2G. After 48 h, the culture medium was collected to harvest virus particles. NCI-H1299 and NCI-H1975 cells were infected with lentivirus and selected by puromycin or G418 to generate stable cell lines.

### Colony formation assay

NCI-H1299 and NCI-H1975 cells were seeded into 6-well plates at a density of 2000 cells per well, and cultured with complete medium, followed by treatment with DMSO or indicated concentration of JNK-IN-8 or/with V9302. After 12 days, cells were fixed with 4% PMF and stained by 0.1% crystal violet solution. Stained colonies were imaged and quantitated.

### Glutamine uptake and glutamate production

Glutamine uptake and glutamate production were measured with Glutamine Assay Kit (#KA1627, Abnova) and Glutamate Assay Kit (#MAK004-1KT, Sigma) following the manufacturer’s instructions. Cells were cultured with 2 mM glutamine with 10% FBS. After 24 h, Cells were calculated and glutamine uptake was calculated by deducting the measured glutamine concentration in the medium from the original glutamine concentration. All values were normalized according to the cell number.

### Western blot and immunoprecipitation

Total protein was collected using RIPA Lysis buffer (#P0013B, Beyotime) supplemented with protease inhibitor and phosphatase inhibitor. Then, the extracted protein was electrophoresed and transferred onto PVDF (#ISEQ00010, Merck Millipore) membrane. After incubation with the corresponding primary and secondary antibodies, protein signaling was visualized using the enhanced chemiluminescence method (#SQ202L, EpiZyme). The antibodies used in this study are as follows: anti-ASCT2 (#A23156, ABclonal; #8057S, Cell Signaling Technology; #ab237704, Abcam), anti-Flag (#AE063, ABclonal), anti-MYC (#AE070, ABclonal), anti-HA(#AE105, ABclonal), anti-JNK1/2/3 (#A22376, ABclonal), anti-Phospho-JNK (#4668S, Cell Signaling Technology; #AP0631, ABclonal), anti-ZDHHC14 (#PA5-61135, Thermo Fisher Scientific), anti-ABHD17B (N/A, PTM BIO), anti-pan Phospho-Serine/Threonine (#AP1475, ABclonal) and anti-Actin (#60004-1-Ig, Proteintech). For Co-IP assays, cells were lysis in NP40 Lysis Buffer (#P0013F, Beyotime) supplemented with protease inhibitor and phosphatase inhibitor. Then, the lysates were mixed with anti-Flag, anti-Myc, or anti-HA magnetic beads (#AE061, ABclonal; #HY-K0201, # HY-K0206, MCE) at 4 °C for 4 h and washed with NP40 Lysis Buffer three times, followed by western blot.

### Alkyne-azide click chemistry experiments

Alkyne-azide click chemistry experiments protocols were performed according to the published procedure^31^. Briefly, cells were cultured with 50 μM Palmitic Acid Alkyne (ALK-14) overnight and collected in 1.0% NP40 lysis buffer (50 mM Tris-HCl, pH 8.0, 150 mM NaCl, 1.0% NP40, 10% Glycerol) with protease inhibitor (Roche). After sonication, the supernatant was collected after centrifugation at 14,000 rpm for 15 min at 4 °C. The target protein was purified with anti-Flag Magnetic beads and washed with IP wash buffer. The beads were suspended in 50 μl PBS. Click chemistry reaction was performed in the following order: 1 μl TAMRA azide (4 mM), 1.2 μl tris ((1-benzyl-1H-1,2,3-triazol-4-yl) methyl) amine (10 mM), 1 μl CuSO4 (40 mM), 1 μl tris (2-carboxyethyl) phosphine HCl (40 mM). The reaction mixtures were mixed thoroughly and incubated for 1 h, protected from light at RT. Then, the beads were washed eight times with PBS and heated at 98 °C for 10 min. Half of the sample was treated with final concentration of 0.5 M hydroxylamine and heating for 98 °C for another 10 min to remove S-palmitoylation. The samples were separated in 10% SDS-PAGE gels, and fluorescence was detected using Biorad Gel Imaging Systems. After detection, the gels were silver-stained to check for protein loading.

### RNA preparation and real-time quantitative reverse transcription (RT-PCR)

Total RNA from lung cancer cells treated with 2-BP was isolated using AG RNAex Pro Reagent (#AG21102, Accurate Biotechnology (Hunan) Co., Ltd, Changsha, China) according to the manufacturer’s instructions. The extracted total RNA was subjected to cDNA synthesis using Evo M-MLV RT Master Mix for qPCR (#AG11706, Accurate Biotechnology (Hunan) Co., Ltd, Changsha, China). Specific quantitative RT-PCR experiments were performed using SYBR Green Premix Pro Taq HS qPCR Kit (#AG11701, Accurate Biotechnology (Hunan) Co., Ltd, Changsha, China) following manufacturer’s protocol. Gene expression level was normalized to actin level in respective samples as an internal control, and analyses were conducted in triplicate. The sequences of the primers used for RT-PCR are listed as follows: ASCT2 (forward: 5′-CATCGTCTTTGGTGTGGCG-3′, reverse:5′-CACAGGGGCGTACCACATG-3′); Actin (forward: 5′-CATGTACGTTGCTATCCAGGC-3′, reverse:5′-CTCCTTAATGTCACGCACGAT-3′).

### Mass spectrometry

To verified the proteins interacts with ASCT2, NCI-H1975 cell was overexpressed with Flag tagged WT or mutate ASCT2 and performed Co-IP assays. The proteomics was performed by Beijing Qinglian Biotech Co., Ltd. Briefly, a 100 μg aliquot of extracted proteins was digest by trypsin. Then, nanoflow LC-MS/MS analysis of tryptic peptides was conducted on a quadrupole Orbitrap mass spectrometer (Orbitrap ExplorisTM 480, Thermo Fisher Scientific, Bremen, Germany) coupled to an EASY nLC 1200 ultra-high-pressure system (Thermo Fisher Scientific) via a nano-electrospray ion source. 500 ng of peptides were loaded on a 25 cm column (150 μm inner diameter, packed using ReproSil-Pur C18-AQ 1.9-μm silica beads; Beijing Qinglian Biotech Co., Ltd, Beijing, China). Peptides were eluted over a 60 min gradient at a flowrate of 600 nL/min, using 80% Acetonitrile, 0.1% Formic acid (Buffer B) going from 8% to 12% over 5 min, to 30% over 30 min, then to 40% over 9 min and to 95% over 1 min, and holding it at 95% for 15 min. Spectra were acquired with an Orbitrap ExplorisTM 480 Mass Spectrometer (Thermo Fisher Scientific) with FAIMS ProTM Interface (Thermo Fisher Scientific) cycling between CVs of −45 V and −65 V every 1 s. MS1 spectra were acquired at 60,000 resolution with a scan range from 350 to 1200 m/z, normalized AGC target of 300%, and maximum injection time of 50 ms. MS1 precursors with an intensity > 5.0e3, charge state of 2–6, and that matched a precursor envelope fit threshold of 30% at 1.6 m/z fit window were selected for MS2 analysis. Here, ions were isolated in the quadrupole with a 0.7 m/z window, collected to a normalized AGC target of 75% or maximum injection time of 22 ms fragmented with 30 normalized HCD collision energy, and resulting spectra acquired at 45,000 resolution with a first mass of 110 m/z. For proteomics data analysis, all RAW files were analyzed using the Proteome Discoverer suite (version 2.4, Thermo Fisher Scientific).

### Immunofluorescence

Cells were grown on chamber slides overnight and fixed with 4% paraformaldehyde for 15 min at RT, and permeabilized samples with Permeabilization Buffer (#00-8333-56, eBioscience™) for 10 min at RT. After incubating in 5% BSA in PBS for 30 min at RT, samples were incubated with primary antibody at appropriate dilution in PBS overnight at 4 °C. Samples were washed with PBS three times and incubated with fluorochrome-conjugated secondary antibody at appropriate dilution in PBS for 1.5 h at RT in dark. Then, samples were washed with PBS three times and incubated with 1 μg/mL DAPI (#C1002, Beyotime). Finally, samples were mounted with Antifade Medium (#P0126, Beyotime) and observed with fluorescence microscope or confocal microscopy (LSM900, Zeiss). Primary and secondary antibodies for anti-LAMP1 (#A19544, ABclonal), Alexa Fluor™ 488 (#A-11008, Invitrogen), Alexa Fluor™ 555 (#A-21422, Invitrogen) were applied for IF.

### GST-pull down

Human MAPK8 (JNK1) recombinant protein (#PV3319) was purchased from Thermo Fisher Scientific, ZDHHC14 recombinant protein (#CSB-CF811637HU) was purchased from CusaBio. For the in vitro binding assays, 0.2 μg purified GST-tagged ZDHHC14 protein or 0.2 μg purified GST protein was incubated with 0.2 μg purified JNK1 protein in IP lysis (50 mM Tris-HCl, pH 7.5, 150 mM NaCl, 10 mM MgCl_2_, 1 mM DTT, and 1 mM EGTA with protease inhibitor) with glutathione beads (#SM002005, Smart-Lifesciences) for 4 h at 4 °C. After washing with IP lysis three times, the samples were separated by SDS-PAGE and immunoprecipitated with the appropriate antibodies followed by western blot.

### In vitro kinase assay

In vitro kinase assay was performed in reaction buffer (0.3 μg kinase, 3 μg substrate, 50 mM Tris-HCl, pH 7.5, 150 mM NaCl, 10 mM MgCl_2_, 1 mM DTT, 1 mM EGTA, and 200 μM ATP) and incubated for 30 min at RT. For negative control, ATP was not provided in reaction buffer. Then, the samples were separated in 10% SDS-PAGE and stained with Coomassie Brilliant Blue (#P0003, Beyotime). The target band was cut out, and the PTM was identified by LC-MS/MS (Beijing Qinglian Biotech Co., Ltd).

### Palmitoylation LFQ proteomics

Palmitoylation LFQ Proteomics was performed by Shanghai Bioprofile Technology Company. Briefly, Cell samples were dissolved with 200 μL lysis buffer (4% SDS, 150 mM Tris-HCl, pH 8.0). All samples were sonicated, and proteins were extracted by centrifugation. Protein from each sample was precipitated with pre-cooled acetone, and the free cysteines of proteins were blocked by mixing for 12 h at 4 °C in 5 mL total PBS containing 0.5% SDS, 1% Triton X-100 (Sigma-Aldrich), protease inhibitors (Thermo Fisher Scientific), 5 mM EDTA, and 25 mM N-ethylmaleimide (NEM, Thermo Fisher Scientific). The samples were precipitated using chloroform/methanol to remove excess NEM. Proteins were resuspended in 1.5 mL of resuspension buffer (4% SDS, protease inhibitors (Thermo Fisher Scientific), 5 mM EDTA in PBS pH 7.4), and mixed with 3 mL of 1 M hydroxylamine (pH 7.4 with NaOH, Thermo Fisher Scientific), and 500 µL of 4 mM Biotin-HPDP (Thermo Fisher Scientific) in DMSO, and then incubated at 25 °C for 2 h with gentle mixing. Proteins were precipitated again and resuspended in 6 M urea and then diluted six-fold with 50 mM ammonium bicarbonate, and then digested with trypsin at 37 °C for 20 h. The tryptic digested peptides were first desalted with C18 spin column (Thermo Fisher Scientific). The dried peptides were resuspended with 200 µL loading buffer (0.2% SDS, 0.2% Triton X-100, and 500 mM NaCl) and mixed with 100 µL of high-capacity streptavidin beads (Thermo Fisher Scientific) for 2 h at room temperature. Beads were washed three times with 5 mL PBS containing 0.2% SDS, 0.2% Triton X-100, and 500 mM NaCl and then collected after wash with 1 mL PBS twice. The beads were then incubated with 0.2 mL elution buffer (50 mM NH_4_HCO_3_, 10 mM TCEP) for 2 h at room temperature. The eluted peptides were collected and mixed with 50 mM iodoacetamide to block reduced cysteine residues, which indicated the palmitoylation sites. Finally, the peptides were desalted with C18 StageTips and prepared for further LC-MS/MS analysis. The MS data were analyzed for interpretation and protein identification against the Homo sapiens database from Uniprot (downloaded on 07/17/2023, and including 207981 protein sequences). The MS spectra were searched using Proteome Discoverer 2.4 software with mass calibration and parameter optimization enabled.

### Tissue microarray

Tissue microarray containing 75 pairs adjacent and matched NSCLC specimens obtained from NSCLC patients was purchased from Shanghai Outdo Biotech Inc (HBreD140Su03). The study was approved by the Ethics Committee of Shanghai Outdo Biotech Company (SHYJS-CP-2304001). All clinical samples were collected with informed consent under the Health Insurance Portability and Accountability Act (HIPAA) approved protocols.

### Tumorigenesis assay

The animal protocols were approved by the Animal Welfare Committee of Laboratory Animal Center of Ningbo University (Protocol Number: AEWC-NBU20250337). To examine the effect of ZDHHC14 on tumorigenesis, male BALB/c nude mice (5–6 weeks old) were randomized into 3 groups (*n* = 6/group) and injected subcutaneously with 5 × 10⁶ cells stably expressing WT ZDHHC14, mutant ZDHHC14, or vector control suspended in 125 μL PBS. To evaluate the effect of ZDHHC14-mediated ASCT2 Cys39 and Cys48 palmitoylation on tumorigenesis, 2 × 10^6^ cells NCI-H1299 cells co-expressing vector control and WT ASCT2 or ASCT2^C39S/C48S^ mutant, and 2 × 10^6^ cells co-expressing ZDHHC14 and WT ASCT2 or ASCT2^C39S/C48S^ mutant were injected subcutaneously into nude mice. In animal experiments involving drug treatment, male BALB/c nude mice (5–6 weeks old) were injected subcutaneously with 5 × 10^6^ cells. When the tumor size reached approximately 100 mm^3^, the mice were assigned randomly to the following groups: (1) control; (2) V9302 (20 mg/kg, daily); (3) JNK-IN-8 (20 mg/kg, daily); (4) a combination of both agents (V9302 (20 mg/kg, daily), JNK-IN-8 (20 mg/kg, daily)) (*n* = 6 mice per group). For V9302 and JNK-IN-8 administration, mice in the treated groups received intraperitoneal injections. Mice were monitored every 4 days for tumor formation using digital calipers. Tumor volume was calculated as (length × width^2^) /2. After 16 days, mice were euthanized via CO₂ inhalation, and tumors were excised and weighed. Data were analyzed using two-tailed Student’s *t*-test (GraphPad Prism v10), with significance at *p* < 0.05.

### Statistical analysis

All quantitative experiments were repeated a minimum of three times and presented as the mean ± standard deviation (SD) or mean ± standard error of the mean (SEM). Statistical significance between groups was determined using a two-tailed Student’s *t*-test, one-way ANOVA, or two-way ANOVA. For quantitative comparisons in CHX chase assay, band intensities were normalized to the loading control and then to the mean of the first group from the same biological replicate. Data are presented as fold change relative to first group (set as 1.0). Gene expression was assessed using Pearson correlation analysis. Survival curves were generated via the Kaplan-Meier method, and differences were evaluated using the log-rank test. Statistical differences were denoted as follows: nonsignificant (ns), *p* > 0.05; *p < 0.05, **p < 0.01, and ***p < 0.001.

## Supplementary information

Supplementary Information

## Data Availability

The original data generated in this work can be directed to the corresponding author for rational reasons.
