# Supplementary material for: ASCT2 palmitoylation regulated by JNK1-ZDHHC14 axis orchestrates glutamine metabolism and NSCLC progression
Source: Cell Discov. 2026 Feb 24;12:13. doi: 10.1038/s41421-026-00870-z (PMC12929575; doi:10.1038/s41421-026-00870-z)

SUPPLEMENTARY FIGURE LEGENDS

Figure S1. ZDHHC14 promotes ASCT2 palmitoylation and lysosome degradation, related to Figure 1

(a) NCI-H1299 (left) and NCI-H1975 (right) cells were treated with 50 μM palmitic acid at the indicated time and subjected to western blot and RT-PCR experiments.

(b) NCI-H1299 (left) and NCI-H1975 (right) cells were treated with 50 μM 2-BP at the indicated time and subjected to RT-PCR experiments.

(c) ASCT2-HA and ZDHHC family members were co-expressed in HEK293T cells, and their interactions were determined following Co-IP analyses.

(d) HEK293T ASCT2 expression cells were transfected with these indicated ZDHHC family members plasmids, followed by immunoprecipitation, the cysteine palmitoyltransferase activity of these members on ASCT2 was tested by Alkyne-azide click chemistry experiments.

Figure S2. ZDHHC14 promotes ASCT2 palmitoylation and lysosome degradation, related to Figure 1

(a) HEK293T cells were expressed with ASCT2-Flag and/or ZDHHC14-Myc. Cell lysates were immunoprecipitated with Flag or Myc magnetic beads and immunoblotted using indicated antibodies to detect protein interactions.

(b) ASCT2 palmitoylation was detected by immunoprecipitation and Alkyne-azide click chemistry experiments in NCI-H1299 control or ZDHHC14- knockout cells.

(c) NCI-H1299 (top) and NCI-H1975 (bottom) cells with stable empty vector or ZDHHC14 expression were treated with DMSO or 2-BP and subjected to immunoprecipitation and Alkyne-azide click chemistry experiments.

(d, e) For CHX assay, NCI-H1975 cells with stable empty vector, wild-type ZDHHC14-Myc (d) or catalytically inactive mutant ZDHHC14^{C195S}-Myc (e) expression were treated with 50 μg/mL CHX for the indicated time, and then the expression levels of ASCT2 and ZDHHC14-Myc were analyzed by western blot. The band density of ASCT2 was quantified and normalized to Actin (mean $\pm$ SD, n = 3). The p-value was determined by two-way ANOVA.

(f) NCI-H1975 (left) and NCI-H1299 (right) cells were transfected with stable empty vector, wild-type ZDHHC14-Myc or catalytically inactive mutant ZDHHC14^{C195S}-Myc, and the expression level of ASCT2 and ZDHHC14-Myc was measured by western blot.

(g) Representative immunofluorescence pictures showing the co-localization of endogenous ASCT2 and LAMP1 in NCI-H1975 cells treated with BafA1, with statistical results shown on the right. Values are means $\pm$ SD from five images. The p-value was determined by two-tailed Student's t-test. DAPI, nucleus. Scale bar = 10 μm.

Figure S3. Cys39 and Cys48 sites of ASCT2 palmitoylation are mainly involved in lysosomal-mediated protein degradation, related to Figure 2

(a) LC-MS/MS spectra of representative ASCT2 peptides carrying palmitoylated Cys39.

(b) ASCT2 palmitoylation mediated by ZDHHC9-Myc was assessed by immunoprecipitation and alkyne–azide click chemistry in HEK293T cells expressing wild-type or C39S-C48S mutant ASCT2.

(c) For CHX assay, NCI-H1299 cells with stable WT-ASCT2 or different ASCT2 mutants expression were treated with 50 μg/ml CHX for the indicated time, and then the expression level of ASCT2 was analyzed by western blot. The band density of ASCT2 was quantified and normalized to Actin (mean ± SD, n = 3). The p-value was determined by two-way ANOVA.

(d, e) HEK293T cells were co-transfected with the indicated plasmids, and ubiquitination of ASCT2 was measured by ubiquitination assay following treatment with MG132 (d) and BafA1(e).

Figure S4. ABHD17B depalmitoylates ASCT2 and promotes ASCT2 stabilization, related to Figure 3

(a) Expression of ASCT2 and ABHD17B was analyzed by western blot in NCI-H1299 cells with stable knockdown of ABHD17B expression (left) or ABHD17B overexpression (right).

(b) NCI-H1299 cells with stable empty vector or ABHD17B expression were treated with 50 μg/ml CHX for the indicated time, the expression level of ASCT2 was tested by western blot. The band density of ASCT2 was quantified and normalized to Actin (mean ± SD, n = 3). The p-value was determined by two-way ANOVA.

(c, d) HEK293T cells expressing empty vector or ABHD17B were co-transfected with the indicated plasmids, and ubiquitination of ASCT2 was measured by ubiquitination assay with MG132 (c) and BafA1(d) treatment.

(e) Trim27 expression in normal and NSCLC tumor tissues was analyzed in the TCGA dataset.

(f) Kaplan-Meier survival analysis of Trim27 for NSCLC patients in an aggregate dataset.

(g) HEK293T cells expressing empty vector or Trim27 were co-transfected with the indicated plasmids, and ubiquitination of ASCT2 was measured by ubiquitination assay following treatment with BafA1.

Figure S5. Glutamine deprivation inhibits ASCT2 palmitoylation through JNK pathway, related to Figure 4

(a) The effect of glutamine deprivation on ASCT2 Palmitoylation in NCI-H1299 cells was examined by immunoprecipitation and Alkyne-azide click chemistry experiments.

(b) ASCT2 and ABHD17B expressing NCI-H1975 cells treated with BafA1 were cultured with or without

glutamine for 12h, their interactions were determined following immunoprecipitation analyses.

(c) NCI-H1975 cells were cultured in the medium with or without glutamine and treated with addition of 50 μg/ml CHX, harvested at indicated time for western blot. The band density of ZDHHC14 was quantified and normalized to Actin (mean ± SD, n=3). The p-value was determined by two-way ANOVA.

(d) Expression of ASCT2 and ZDHHC14 was analyzed by western blot in NCI-H1975 cells cultured in the medium with or without glutamine and treated with these indicated signaling pathway inhibitors (5 μM LY294002 and 5 μM AZD5363 for inhibiting AKT, 2 μM Dorsomorphin dihydrochloride for inhibiting AMPK, 5 μM Ravoxertinib and 5 μM SCH772984 for inhibiting ERK, 5 μM JNK-IN-8 for inhibiting JNK, 5 μM Adezmapimod and 5 μM SB-202190 for inhibiting p38, and 10 μM MHY1485 for activating mTOR) for 12 h.

(e) NCI-H1975 cells were treated with DMSO, 10 μM MG132, 0.1 μM carf, 25 μM CQ or 0.1 μM BafA1 combined with 0.5 μM Anisomycin for 6 h. ZDHHC14 expression was measured by western blot.

(f, g) NCI-H1975 cells expressing ASCT2-WT (f) or ASCT2C$^{39S\text{-}C48S}$ mutant (g) were treated with 0.5 μM Anisomycin at the indicated concentrations for 12 h and subjected to western blot with ASCT2, ZDHHC14, JNK1/2/3, Phospho-JNK1/2/3 and Actin antibodies.

(h) NCI-H1299 cells expressing ASCT2-Flag were treated with DMSO or JNK-IN-8 and cultured with or without glutamine for 12 h, ASCT2 palmitoylation was assessed by IP and Alkyne–azide click chemistry.

Figure S6. JNK1-mediated phosphorylation stabilizes ASCT2 by triggering ZDHHC14 degradation, related to Figure 5

(a) Protein-protein docking analysis examined a direct protein-protein interaction between ZDHHC14 and JNK1.

(b) NCI-H1299 ZDHHC14-knockout cells were transfected with ZDHHC14-Myc for Co-IP assays using anti-Myc antibodies and then analyzed by western blot.

(c) Schematic of the in vitro kinase assay combined with liquid chromatography tandem mass spectrometry (LC-MS/MS) (top). Table showing the phosphorylated peptides identified by LC-MS/MS (bottom).

(d) LC-MS/MS spectra of representative ZDHHC14 peptides carrying phosphorylated Thr124 and Ser455

(e) Wild-type ZDHHC14 or phosphorylation mutants expressing NCI-H1299 (top) and NCI-H1975 (bottom) cells were cultured with or without glutamine, the expression level of ZDHHC14 was measured by western blot.

(f) For CHX chase assay, ZDHHC14-WT or ZDHHC14^{T440A} was expressed in NCI-H1975 cells, and then the cells were treated with 50 μg/mL CHX for the indicated time, the expression level of ZDHHC14 was analyzed by western blot. The band density of ZDHHC14 was quantified and normalized to Actin (mean ± SD, n=3). The p-value was determined by two-way ANOVA.

Figure S7. JNK1-mediated phosphorylation stabilizes ASCT2 by triggering ZDHHC14 degradation, related to Figure 5

(a) NCI-H1299 ZDHHC14-knockout cells expressing with ZDHHC14-WT or ZDHHC14^{T440A} were treated with the indicated conditions, ZDHHC14 phosphorylation was measured by immunoprecipitation.

(b) NCI-H1299 ZDHHC14-knockout cells were transfected with ZDHHC14-WT or ZDHHC14^{T440A} plasmid, western blot analyzed the effect of JNK inhibitor on ASCT2 and ZDHHC14 stabilization.

(c) NCI-H1975 cells co-expressing with ASCT2-Flag and ZDHHC14-WT/ZDHHC14^{T440A} mutant were cultured in the medium with or without glutamine for 12 h, immunoprecipitation and Alkyne-azide click chemistry experiments examined the palmitoylation level of ASCT2.

(d) NCI-H1299 (left) and NCI-H1975 (right) cells expressing ZDHHC14-WT or ZDHHC14^{T440A} were treated with Anisomycin and Alk14 for 8 h, and the palmitoylation level of ASCT2 was analyzed by immunoprecipitation and Alkyne-azide click chemistry experiments.

Figure S8. ZDHHC14-ASCT2 axis regulates glutamine metabolism and tumorigenesis in NSCLC, related to Figure 6

(a) ASCT2 (left) and ZDHHC14 (right) expression in normal and NSCLC tissues was analyzed in TCGA datasets. The p-value was determined by Wilcoxon rank sum test.

(b) Kaplan-Meier survival analysis of ASCT2 (left) and ZDHHC14 (right) for NSCLC patients in an aggregate dataset.

(c) Box plots indicated ASCT2 (left) and ZDHHC14 (right) expression in different clinical stages of lung adenocarcinoma (LUAD) and lung squamous cell carcinoma (LUSC) tissues from TCGA dataset. The significance level was determined by using the Wilcoxon rank sum test.

(d) Representative images of ASCT2, ZDHHC14 and p-JNK consecutive IHC staining in 75 pairs adjacent and matched NSCLC tissues. Scale bar = 1.5 mm. Linear regression was used to plot the best-fit line, and the Spearman correlation test was used to determine the p value. $p < 0.05$ was deemed statistically significant.

Figure S9. Synergistic anti-cancer activity of the combination of V9302 and JNKi in NSCLC, related to Figure 7

(a, b) Glutamine uptake and glutamate production in NCI-H1975 (left) and NCI-H1299 (right) cells treated with 1μM ABD957, 2.5μM V9302, alone or in combination for 24h were measured by assays for glutamine uptake (a) and glutamate production (b) detection (mean ± SD in three separate experiments). The p-value was

determined by one-way ANOVA.

(c, d) Glutamine uptake and glutamate production in NCI-H1975 (left) and NCI-H1299 (right) cells treated with 0.25 μM JNK-IN-8, 2.5μM V9302, alone or in combination for 24h were measured by assays for glutamine uptake (c) and glutamate production (d) detection (mean ± SD in three separate experiments). The p-value was determined by one-way ANOVA.

(e, f) 72h dose-response curves for JNK-IN-8 treatment in NCI-H1299 (left) and NCI-H1975 (right) cells (e). Table showing the IC50 values of JNK-IN-8 in NCI-H1299 and NCI-H1975 cells (f).

(g) Pictures of the mouse model treated with JNK-IN-8, V9302, alone or in combination in vivo.

(h) A proposed model to illustrate mechanisms of ASCT2 palmitoylation.

Figure S1 ZDHHC14 promotes palmitoylation of ASCT2 and lysosome degradation

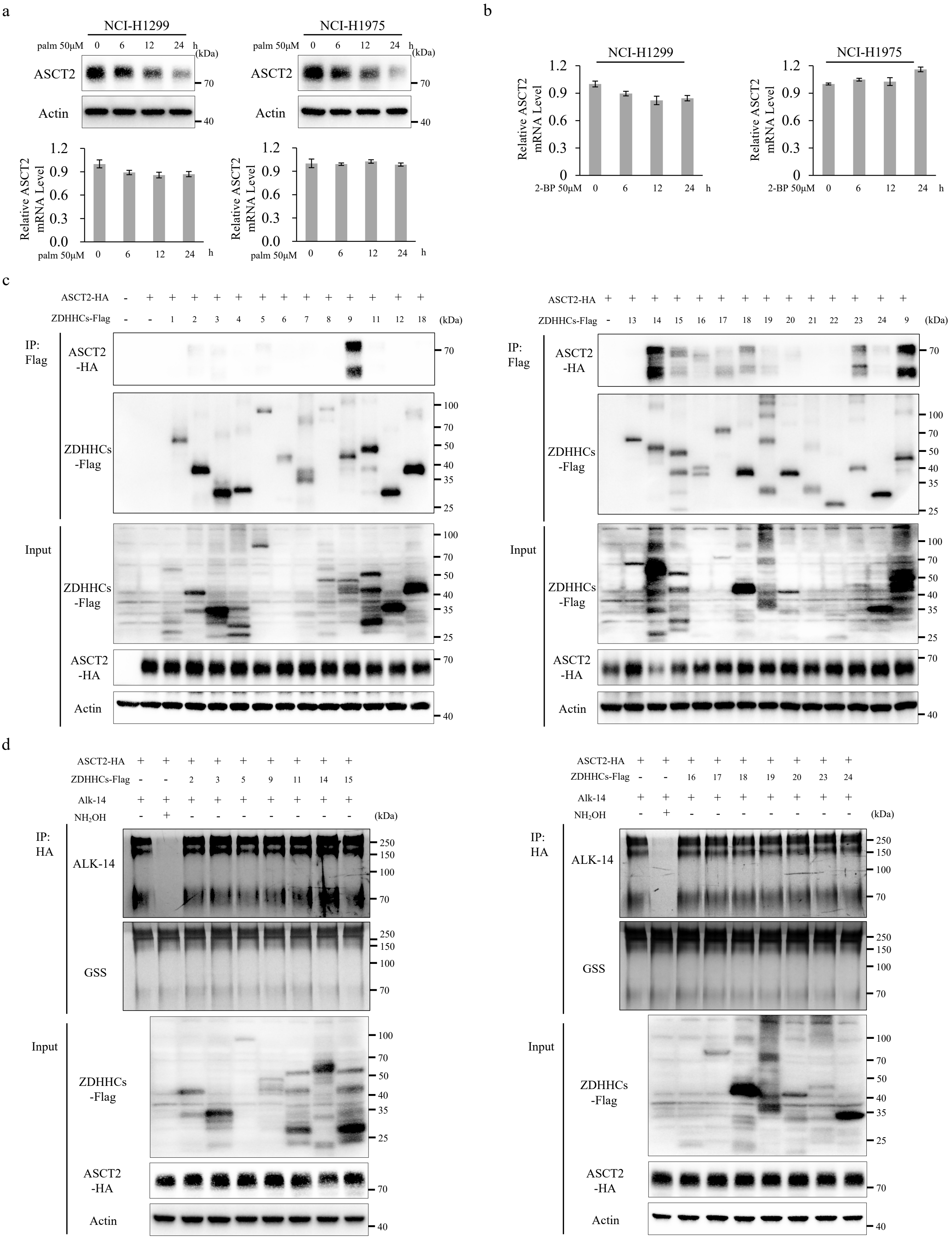

Figure S2 ZDHHC14 promotes palmitoylation of ASCT2 and lysosome degradation

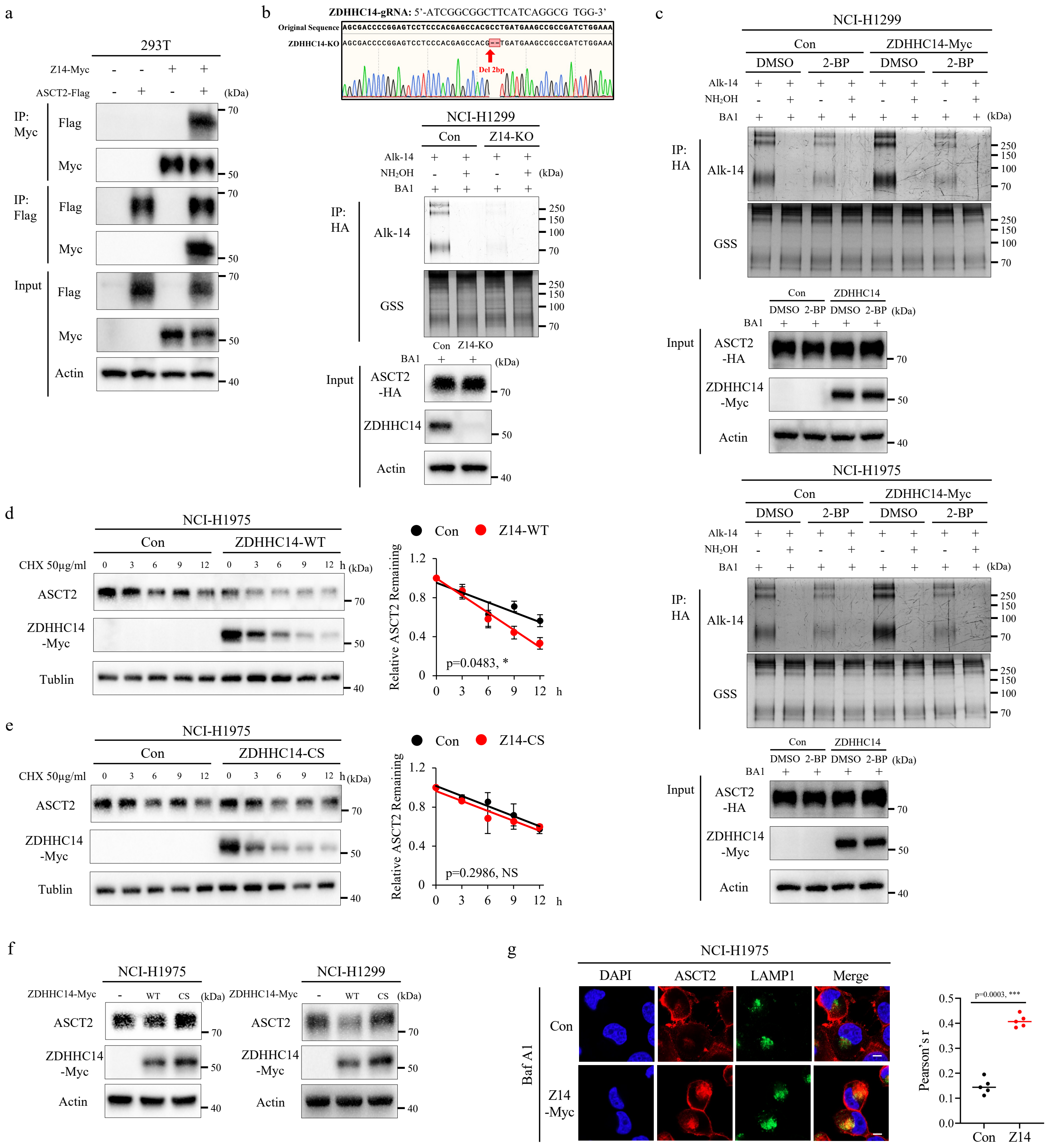

Figure S3 Cys39 and Cys48 sites of ASCT2 palmitoylation are mainly involved in lysosomal-mediated protein degradation

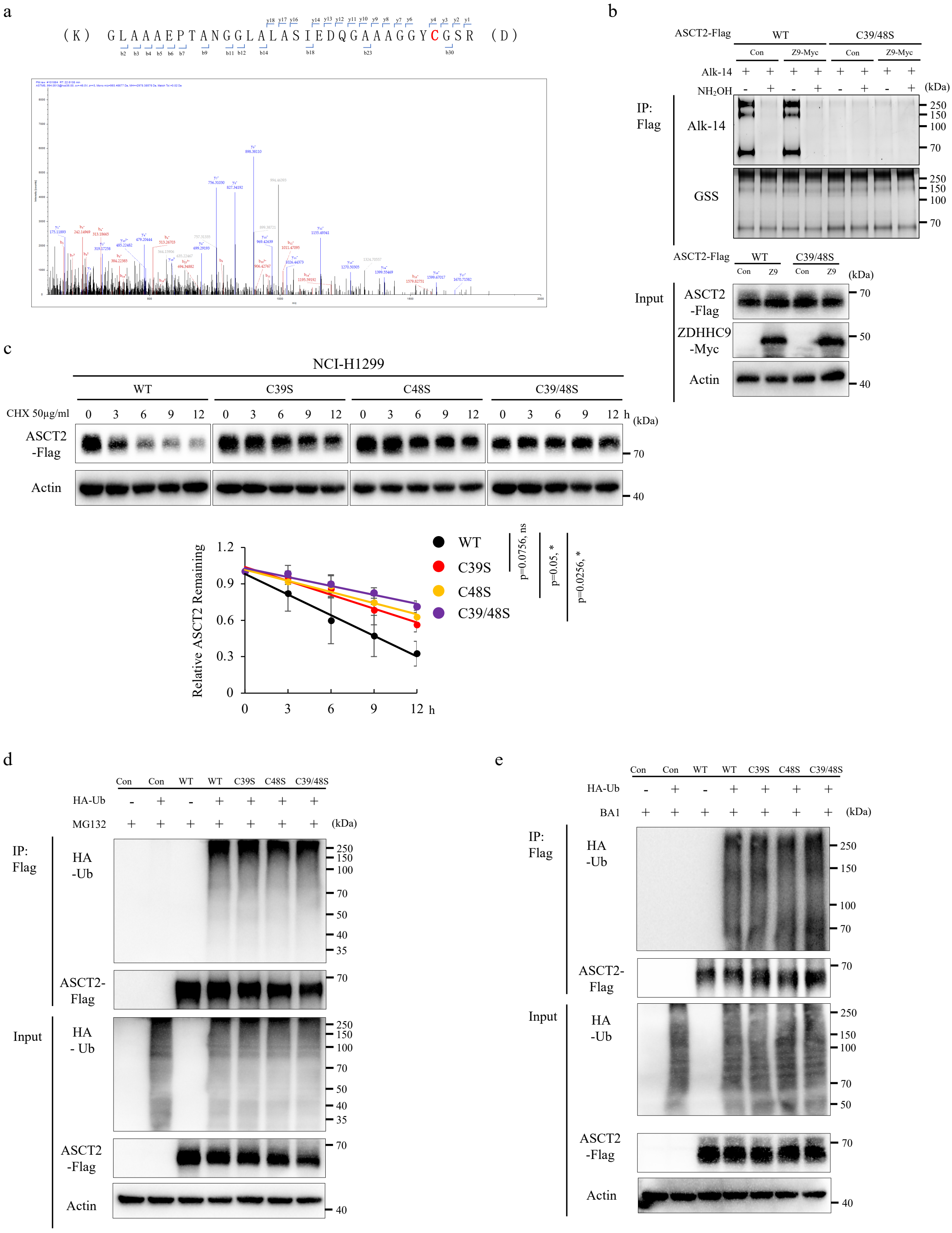

Figure S4 ABHD17B depalmitoylates ASCT2 and promotes ASCT2 stabilization

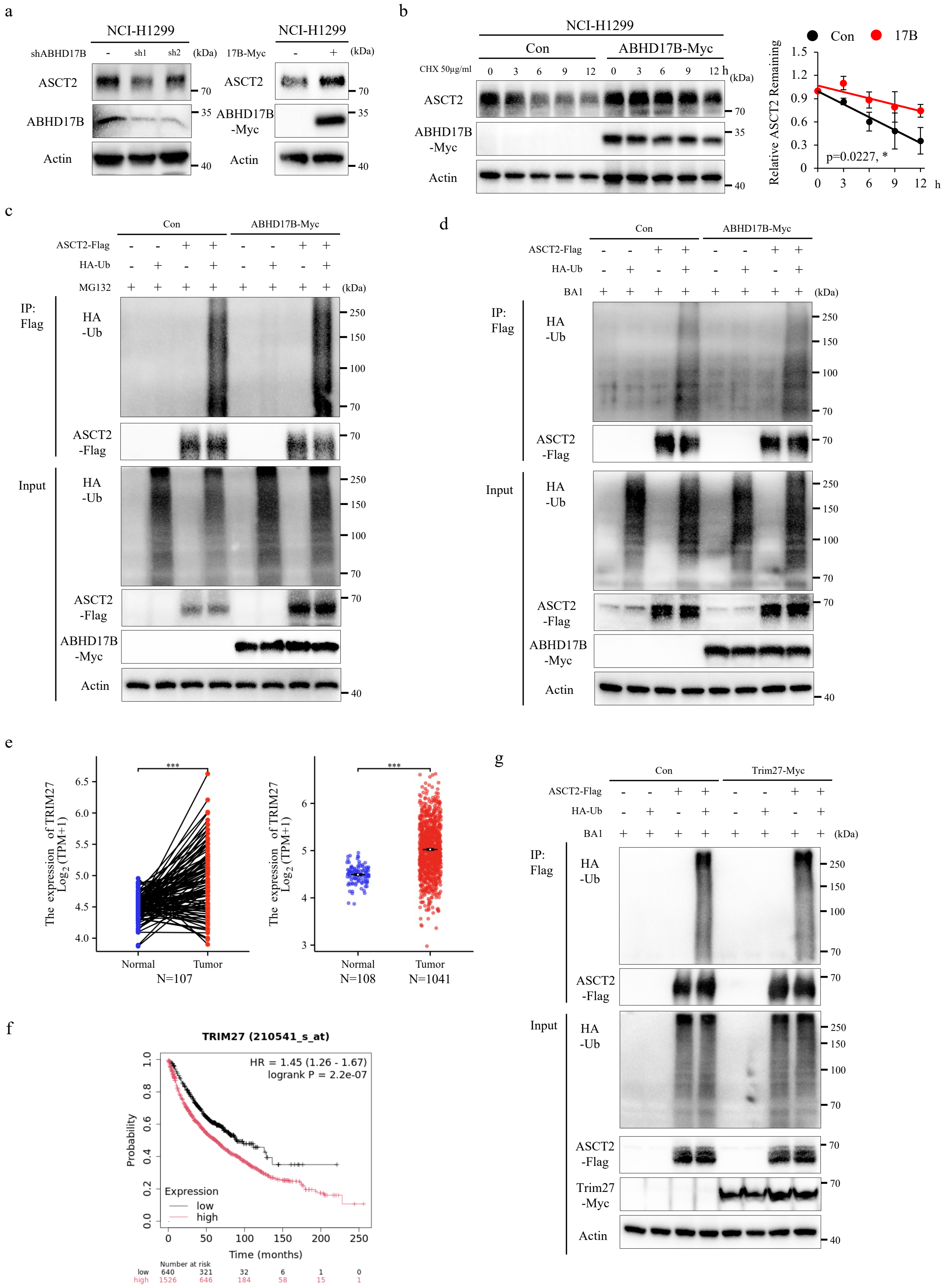

Figure S5 Glutamine deprivation inhibits ASCT2 palmitoylation through JNK pathway

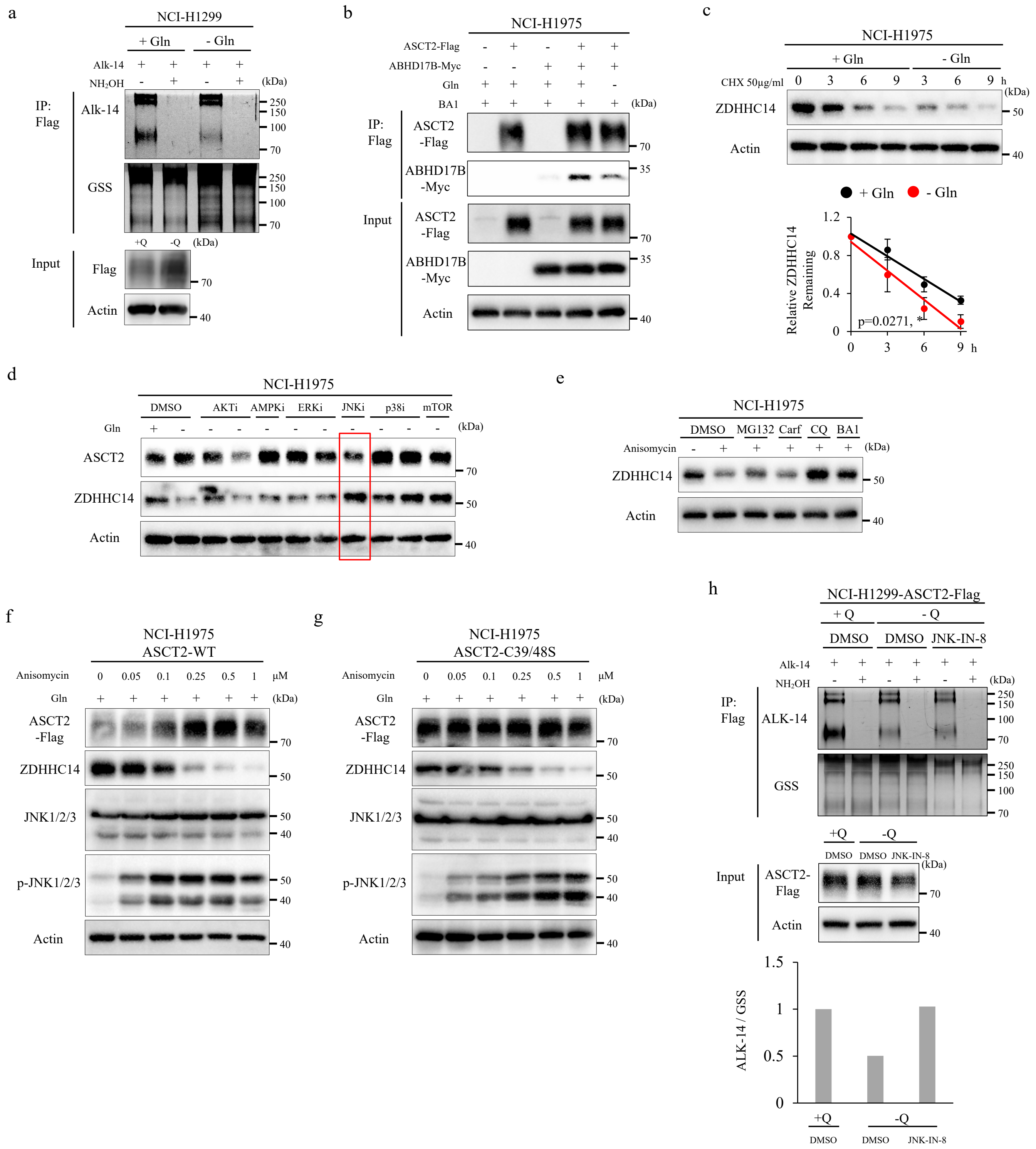

Figure S6 JNK1-mediated phosphorylation stabilizes ASCT2 by triggering ZDHHC14 degradation

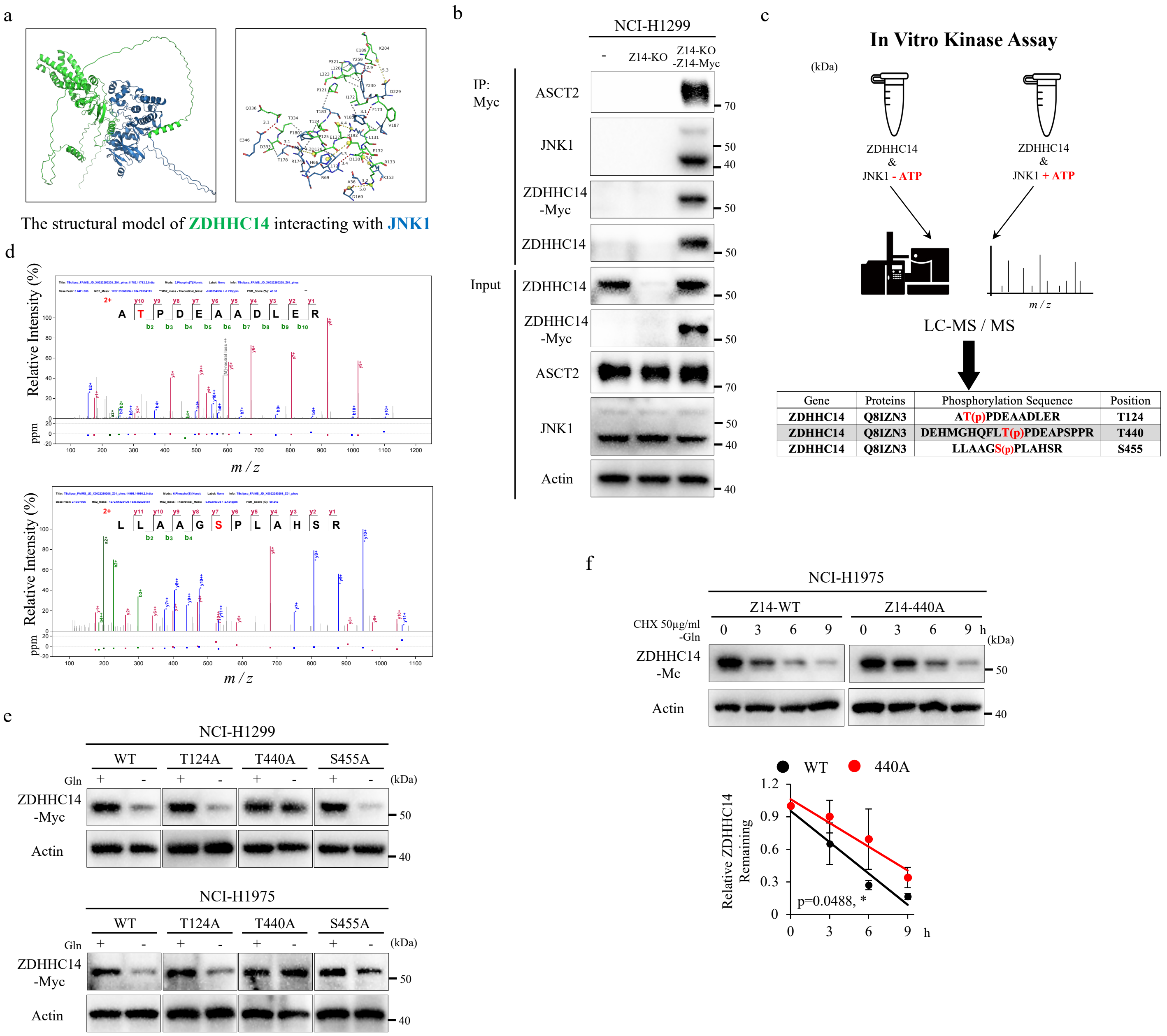

Gene	Proteins	Phosphorylation Sequence	Position
ZDHHC14	Q8IZN3	AT(p)PDEAADLER	T124
ZDHHC14	Q8IZN3	DEHMGHQFLT(p)PDEAPSPPR	T440
ZDHHC14	Q8IZN3	LLAAGS(p)PLAHSR	S455

Figure S7 JNK1-mediated phosphorylation stabilizes ASCT2 by triggering ZDHHC14 degradation

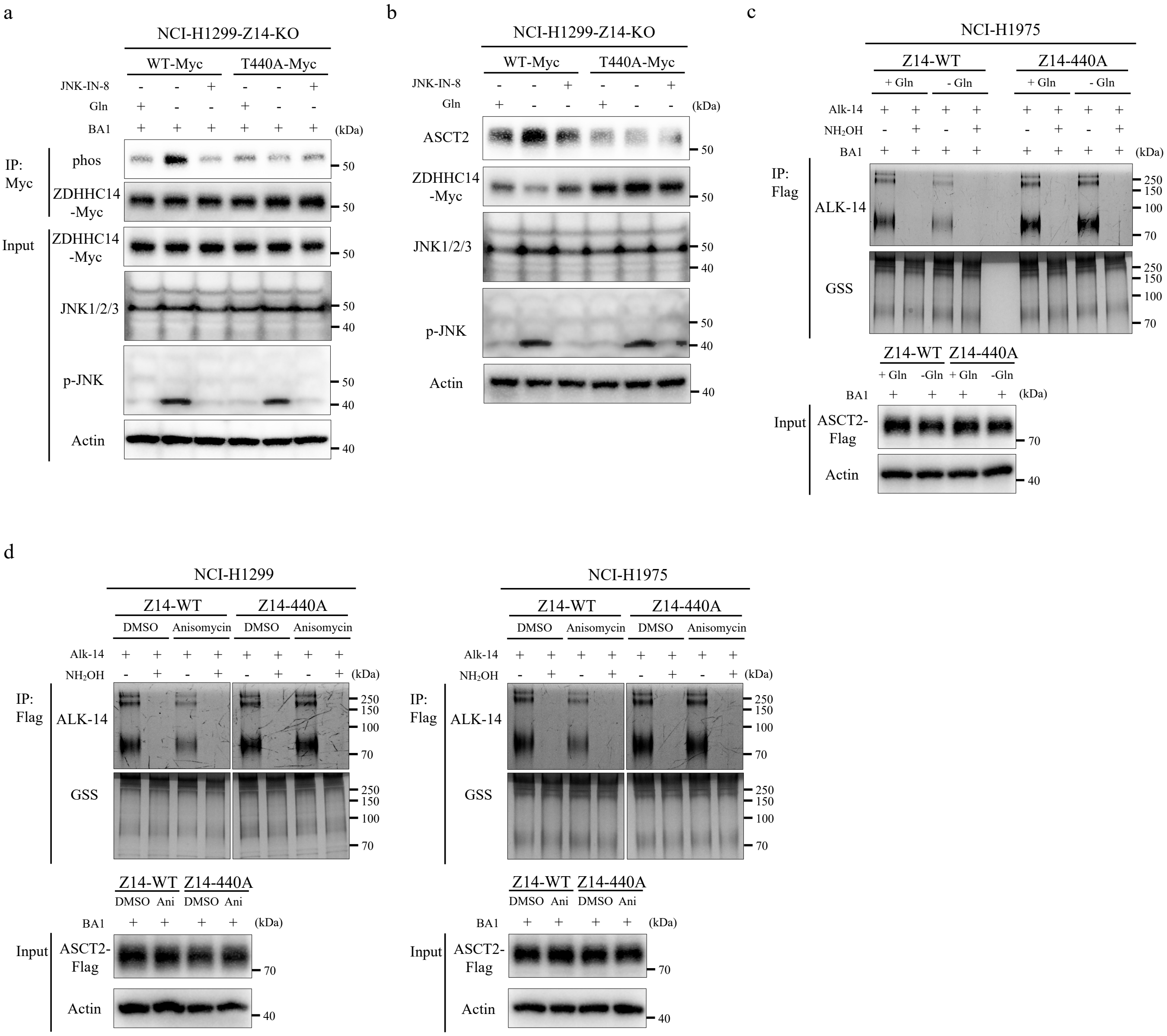

Figure S8 ZDHHC14-ASCT2 axis regulates glutamine metabolism and tumorigenesis in NSCLC

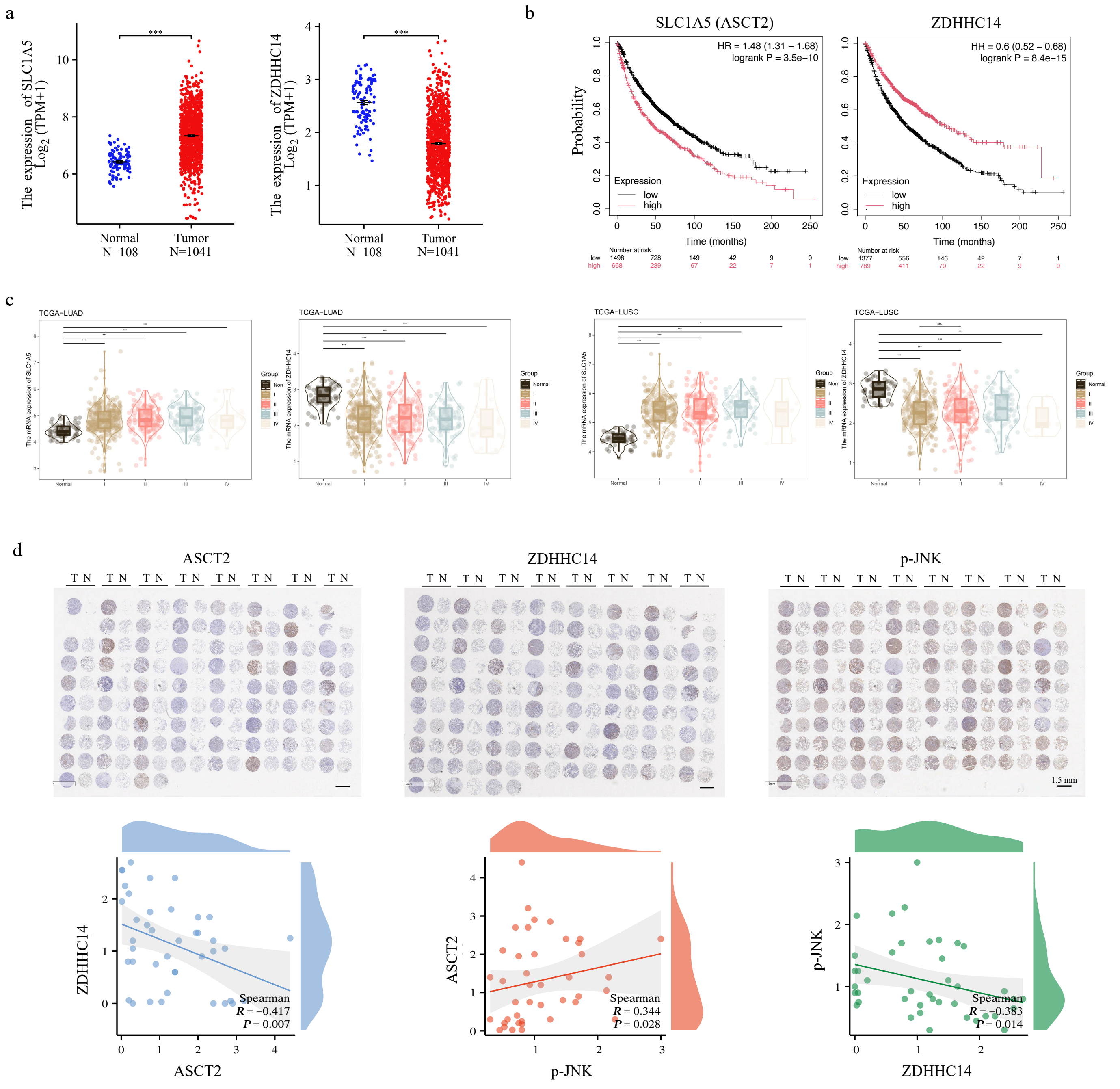

Figure S9 Synergistic anti-cancer activity of the combination of V9302 and JNKi in NSCLC

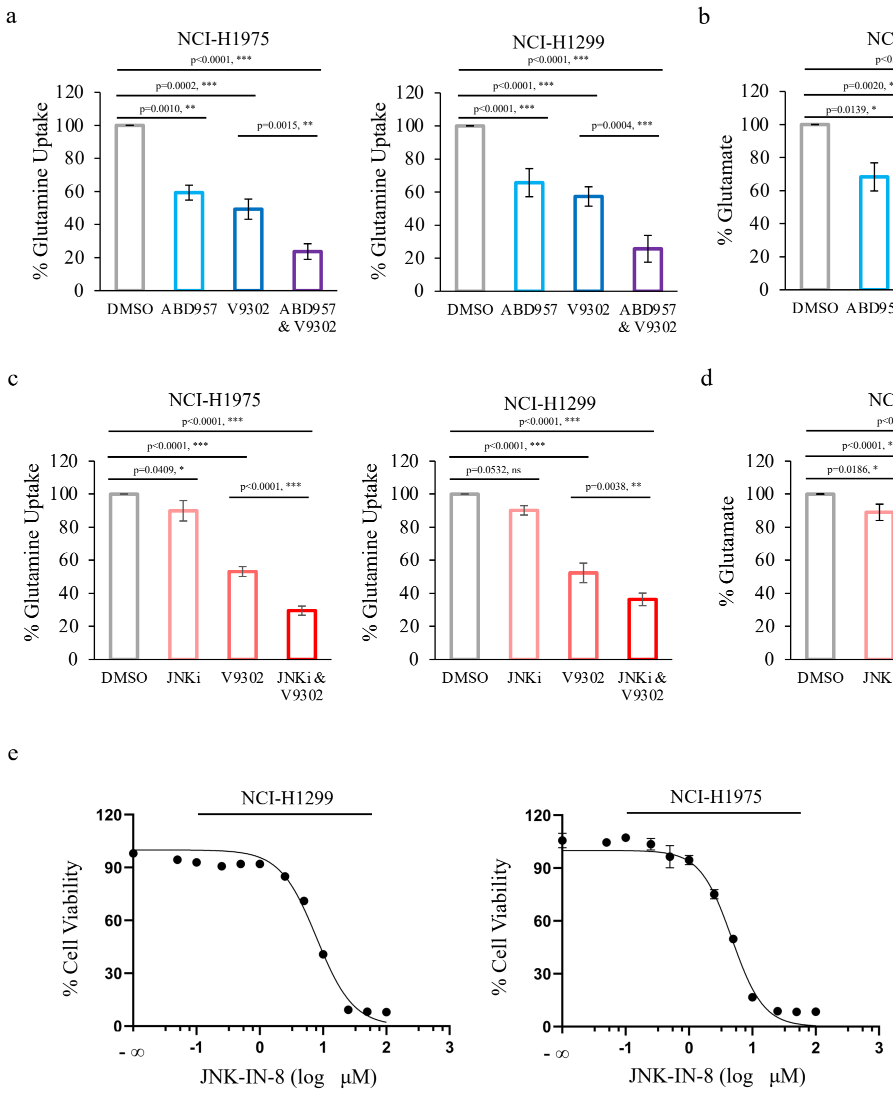

f

	NCI-H1299 JNK-IN-8	NCI-H1975 JNK-IN-8
IC50 (µM)	7.930	4.749

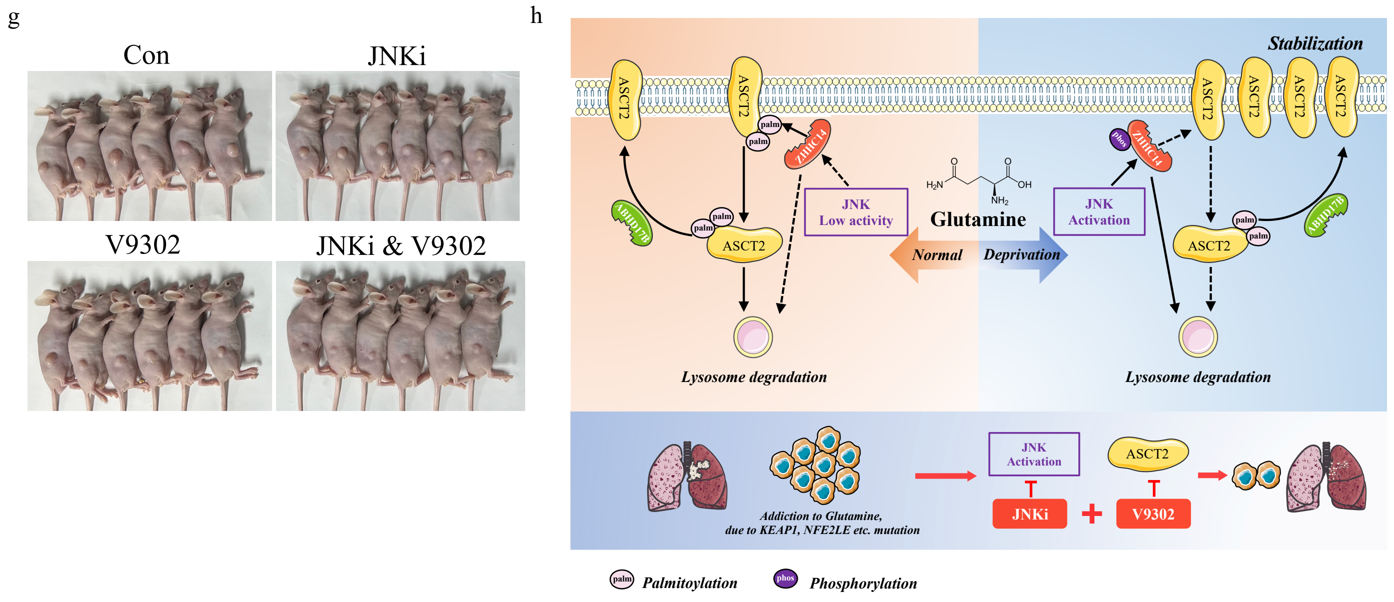

Supplement: Supplementary file 1 — Supplementary Information [file 41421_2026_870_MOESM1_ESM.pdf]
